# MR-pheWAS with stratification and interaction: Searching for the causal effects of smoking heaviness identified an effect on facial aging

**DOI:** 10.1371/journal.pgen.1008353

**Published:** 2019-10-31

**Authors:** Louise A. C. Millard, Marcus R. Munafò, Kate Tilling, Robyn E. Wootton, George Davey Smith

**Affiliations:** 1 MRC Integrative Epidemiology Unit at the University of Bristol, Bristol, United Kingdom; 2 Department of Population Health Sciences, Bristol Medical School, University of Bristol, Bristol, United Kingdom; 3 Intelligent Systems Laboratory, Department of Computer Science, University of Bristol, Bristol, United Kingdom; 4 UK Centre for Tobacco and Alcohol Studies, School of Experimental Psychology, University of Bristol, Bristol, United Kingdom; Cornell University, UNITED STATES

## Abstract

Mendelian randomization (MR) is an established approach to evaluate the effect of an exposure on an outcome. The gene-by-environment (GxE) study design can be used to determine whether the genetic instrument affects the outcome through pathways other than via the exposure of interest (horizontal pleiotropy). MR phenome-wide association studies (MR-pheWAS) search for the effects of an exposure, and can be conducted in UK Biobank using the PHESANT package. In this proof-of-principle study, we introduce the novel GxE MR-pheWAS approach, that combines MR-pheWAS with the use of GxE interactions. This method aims to identify the presence of effects of an exposure while simultaneously investigating horizontal pleiotropy. We systematically test for the presence of causal effects of smoking heaviness–stratifying on smoking status (ever versus never)–as an exemplar. If a genetic variant is associated with smoking heaviness (but not smoking initiation), and this variant affects an outcome (at least partially) via tobacco intake, we would expect the effect of the variant on the outcome to differ in ever versus never smokers. We used PHESANT to test for the presence of effects of smoking heaviness, instrumented by genetic variant rs16969968, among never and ever smokers respectively, in UK Biobank. We ranked results by the strength of interaction between ever and never smokers. We replicated previously established effects of smoking heaviness, including detrimental effects on lung function. Novel results included a detrimental effect of heavier smoking on facial aging. We have demonstrated how GxE MR-pheWAS can be used to identify potential effects of an exposure, while simultaneously assessing whether results may be biased by horizontal pleiotropy.

## Author summary

Mendelian randomization uses genetic variants associated with an exposure to investigate causality. For instance, a genetic variant that relates to how heavily a person smokes, has been used to test whether smoking causally affects health outcomes. Mendelian randomization is biased if the genetic variant also affects the outcome via other pathways. We exploit additional information–that the effect of heavy smoking only occurs in people who actually smoke–to overcome this problem. By testing associations in ever and never smokers separately we can assess whether the genetic variant affects an outcome via smoking or another pathway. If the effect is at least partially via smoking heaviness, we would expect the effect to differ in ever versus never smokers, and this would suggest that smoking causally influences the outcome. Previous Mendelian randomization studies of smoking heaviness focused on specific outcomes–here we searched for the presence of causal effects of smoking heaviness across over 18 000 traits. We identified previously established effects (e.g. a detrimental effect on lung function) and novel results including a detrimental effect of heavier smoking on facial aging. Our approach can be used to test for the presence of causal effects of other exposures, where the exposure only occurs in known subsets of the population.

## Introduction

Mendelian randomization (MR) is an established approach that uses genetic variants as proxies for a modifiable exposure, to test for the presence of a causal effect of the exposure (or estimate the magnitude of its effect) on a potential outcome [1]. MR is often implemented within an instrumental variable (IV) framework, with a key assumption being that there is no alternative pathway between the genetic instrument and the outcome that is not via the exposure of interest (exclusion restriction). Horizontal pleiotropy [2], where the genetic instrument affects the outcome through pathways not via the exposure, invalidates this assumption. Whilst the exclusion restriction cannot be statistically tested, MR studies can include an investigation of evidence for a violation of this MR assumption [3]. For instance, where several independent genetic instruments exist for an exposure, a difference in the effect estimates may indicate they are acting through different pathways [4–6]. The majority of these approaches are only applicable where there are several genetic variants that proxy for the exposure of interest [3,7].

A design in which the genetic variant is interacted with another variable can provide evidence for violation of the exclusion restriction assumption. The additional variable can be of many forms; not necessarily environmental. For example, an early study interacted a genetic variant related to alcohol with sex in a population where women drank very little, such that an effect of the variant on the outcome in women would suggest that at least some of the effect of the variant on the outcome is not acting through alcohol consumption [8]. Here we report interaction with another factor, and refer to the design henceforth as involving gene-by-environment (GxE) interaction [8–12]. In contrast to traditional GxE studies that aim to identify genetic variants whose effects on an outcome are modified by an environmental factor (or vice-versa) [13], the aim here is to determine whether horizontal pleiotropy may be biasing estimated causal effects [12]. To conduct a GxE MR study, a phenotype is chosen that stratifies the population into two (or more) groups, with different levels of the exposure. In the ideal case the exposure will manifest in one group but not in the other. If the effect of the genetic variant is (at least partly) via the exposure, the estimate of the direct test between the genetic variant and the outcome should vary in proportion to the degree the exposure manifests in each group. Importantly, the genetic instrument should not be associated with the phenotype used to stratify the sample, otherwise estimates may be biased due to conditioning on a collider [14,15]. We also note that, while we focus on horizontal pleiotropy in this work, GxE MR can be used to assess causal effects in the presence of other violations of the exclusion restriction as long as this violation is the same across strata of the modifying factor (see for example S7 Fig).

**Fig 1:**
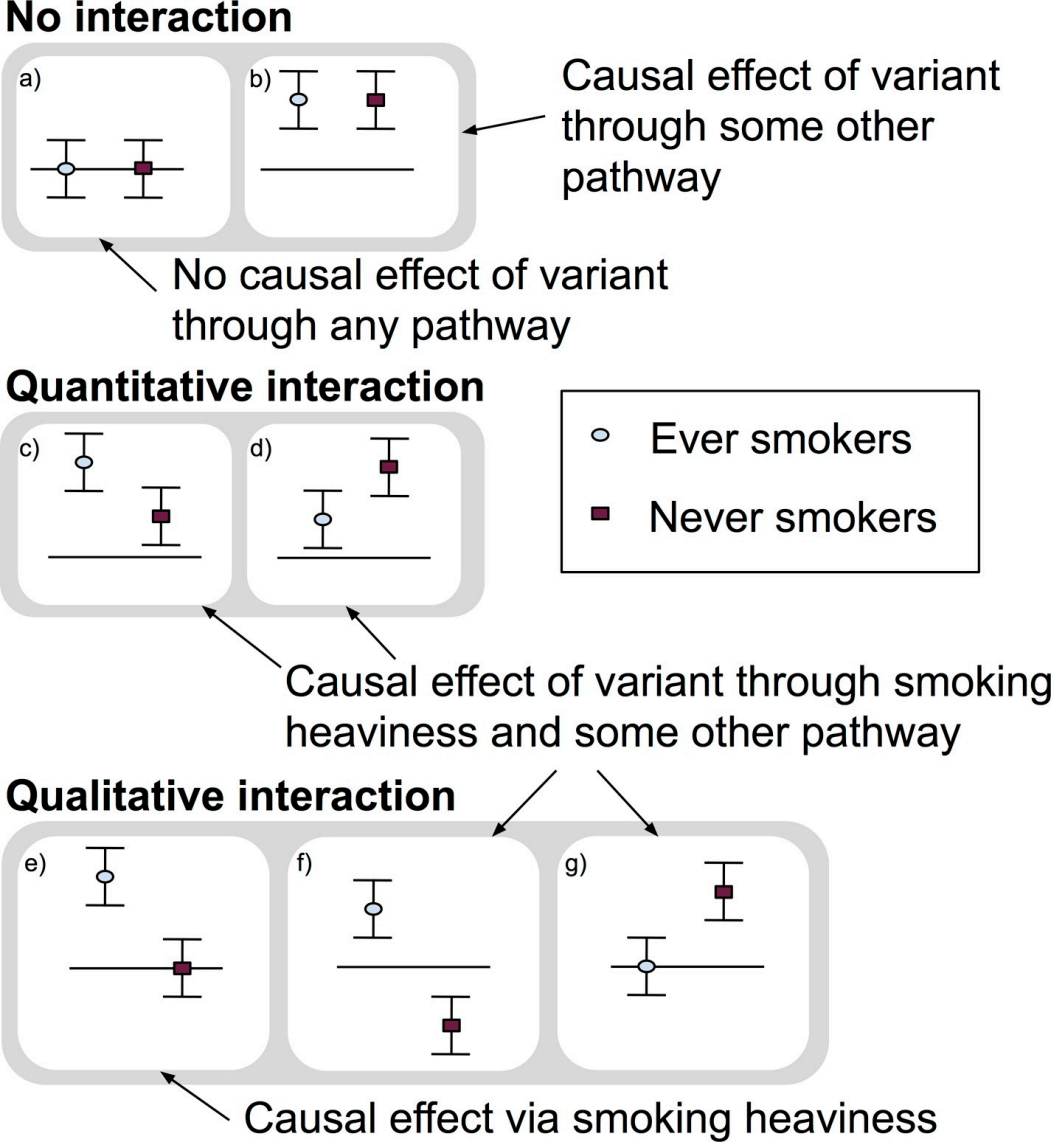
Illustration of possible GxE MR results and their interpretation. This figure illustrates the three broad types of results of a GxE study on smoking heaviness: 1) no interaction between ever and never smokers, 2) a quantitative interaction, where associations are in a consistent direction but one is stronger than the other, or 3) a qualitative interaction, where the effect only occurs in one group or the estimates are in opposite directions, also known as a cross over effect [22]. Both qualitative and quantitative interactions can provide evidence of a causal effect of the genetic variant via smoking heaviness. However, when an effect exists in never smokers the estimate in ever smokers may be comprised of both an effect of the genetic variant through smoking heaviness and an effect through another pathway.

The MR GxE approach has been used to investigate the effect of smoking heaviness on traits such as body mass index (BMI) and depression/anxiety [16–21]. A causal effect of a genetic variant known to affect smoking heaviness should, if the effect is solely via tobacco intake, be seen in participants who are previous or current smokers, but not in participants who have never smoked. Thus, by stratifying on smoking status (ever versus never), we can assess if the smoking heaviness genetic variant is likely to be affecting an outcome via smoking heaviness, or some other pathway (or both). Fig 1 illustrates the three broad types of results of a GxE study on smoking heaviness: 1) no interaction between ever and never smokers, 2) a quantitative interaction, where associations are in a consistent direction but one is stronger than the other, or 3) a qualitative interaction, where the effect only occurs in one group or the estimates are in opposite directions, also known as a cross over effect [22]. Both qualitative and quantitative interactions can provide evidence of a causal effect of the genetic variant via smoking heaviness. However, when an effect exists in never smokers the estimate in ever smokers may be comprised of both an effect of the genetic variant through smoking heaviness and an effect through another pathway.

While MR studies have, to date, been largely performed in a hypothesis-driven manner, it is now possible to perform hypothesis-free MR analyses using the MR phenome-wide association study (MR-pheWAS) approach [5,23]. MR-pheWAS search for the presence of causal effects of an exposure of interest, by testing the direct effect of a genetic instrument on a potentially large set of outcomes. We recently published a software package for performing comprehensive phenome scans in UK Biobank called PHESANT [23]. PHESANT allows researchers to systematically test the association of a trait of interest across over 22 000 phenotypes in UK Biobank, and is the first freely-available tool for performing comprehensive phenome scans. In this paper we combine this hypothesis-free approach with the use of GxE interactions in a novel GxE MR-pheWAS approach. This method enables hypothesis-free searching for the presence of causal effects of an exposure, while simultaneously assessing whether the genetic instrument affects the outcome through other pathways (horizontal pleiotropy). In contrast to hypothesis-driven GxE studies, our hypothesis-free study presented here is intended to be hypothesis-generating–identifying potentially interesting associations to follow-up in independent data. Our method provides evidence about which outcomes are likely to be caused by the exposure, but does not attempt to estimate the magnitude of the identified causal effects. GxE MR-pheWAS can be performed using the PHESANT software. We describe the methods we have developed, compare different options for carrying out the analysis, and perform a proof of principle study, searching for the effects of smoking heaviness. We then discuss the important lessons raised by this first application of the approach.

## Results

Each additional smoking-increasing allele of rs16969968 was associated with a 1.21 [95% confidence interval (CI): 1.19, 1.23] higher odds of being in a higher smoking heaviness category, after adjusting for age, sex and the first 10 genetic principal components, and a 0.98 [95% CI: 0.97, 0.99] lower odds of being an ever (vs never) smoker.

### Results of GxE MR-pheWAS analysis

#### Identified interactions from GxE MR-pheWAS in ever smokers versus never smokers

**Fig 2:**
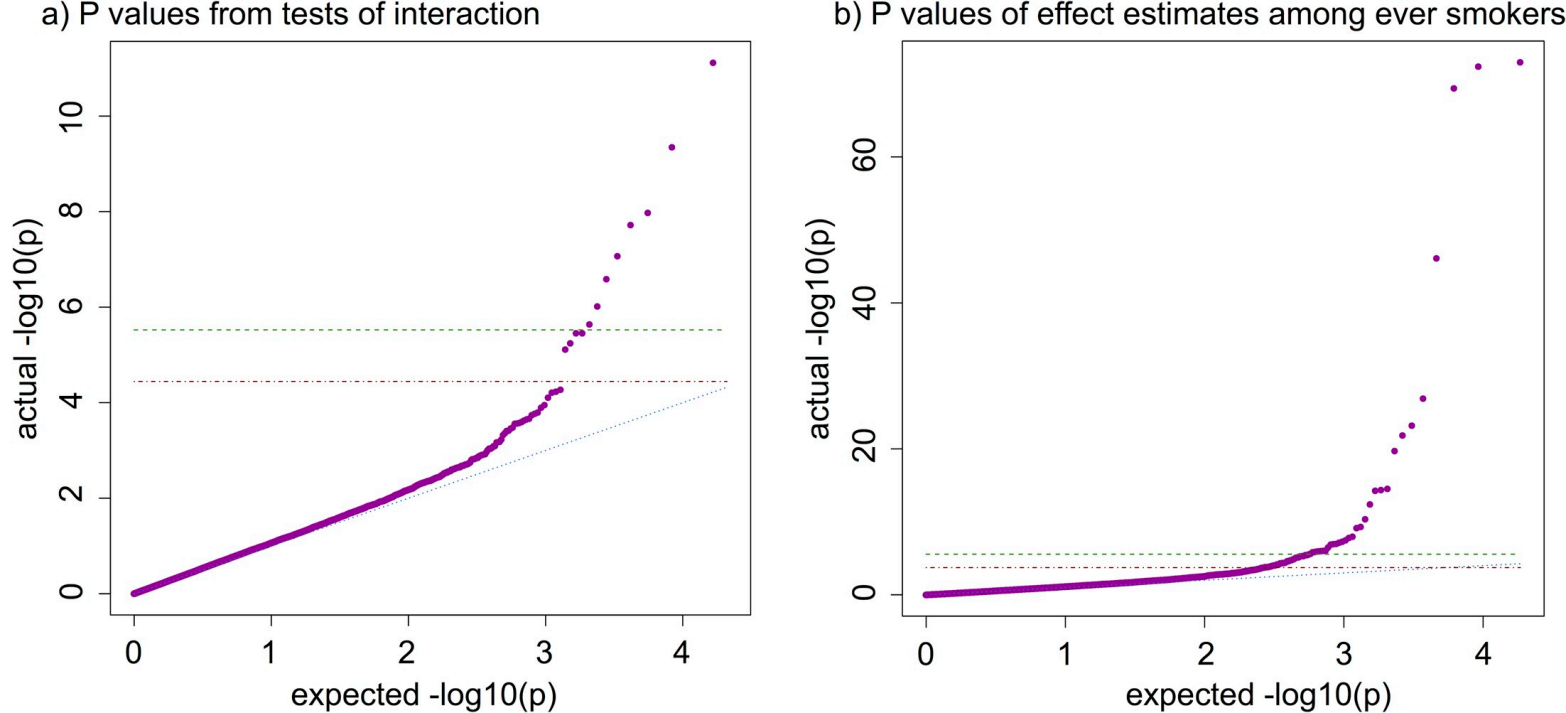
QQ plots of PHESANT MR-pheWAS results. Green dashed: Bonferroni corrected threshold; Red dash-dotted: 5% false discovery rate (FDR) threshold; Blue dotted: Expected = Actual; Purple points: results of tests performed: a) P-values of tests of interaction in GxE MR-pheWAS, and b) P-values of SNP-outcome associations in MR-pheWAS among ever smokers. Multiple testing thresholds: a) Bonferroni threshold: 0.05/16692 = 3.00x10^-6^; 5% FDR threshold: 0.05x12/16692 = 3.59x10^-5^, and b) Bonferroni threshold: 0.05/18513 = 2.70x10^-6^; 5% FDR threshold: 0.05x69/18513 = 1.86x10^-4^.

We performed a GxE MR-pheWAS analysis, ranking on the strength of the interaction between ever versus never smokers (using the P value of Cochran’s Q-test statistic for heterogeneity). Of the 16 692 interactions tested, we identified 8 results with a P value lower than a stringent Bonferroni corrected threshold of 3.00x10^-6^ (0.05/16 692), where an increase in rs16969968 smoking-heaviness increasing allele dosage was associated with worse lung function (3 phenotypes), lower likelihood of being a morning person, and higher blood assay levels (haematocrit percentage, white blood cell count, neutrophil count and haemoglobin concentration) on average, in ever compared with never smokers. We found a further 4 results at a false discovery rate of 5% (using a P value threshold of 0.05x12/16 692 = 3.59x10^-5^) (see S2 Table), where an increase in rs16969968 smoking-heaviness increasing allele dosage was associated with a higher risk of chronic obstructive pulmonary disease, emphysema and cancer diagnoses and greater facial aging, in ever compared with never smokers. A QQ plot is given in Fig 2A.

#### Identified main effects from MR-pheWAS in ever smokers

While our main aim is to identify interactions between the associations in ever versus never smokers (which may suggest a causal effect via smoking heaviness), here we ranked outcomes by the strength of association with rs16969968 in ever smokers to maximize statistical power. This approach relies on the assumption that the combined effect through smoking heaviness and any horizontal pleiotropic effect is not null. For example, if the effects via smoking heaviness and a horizontally pleiotropic pathway are of the same magnitude but in opposite directions then they would cancel out and not be identified in a MR-pheWAS in ever smokers. Under the assumption of no horizontal pleiotropy a 5% FDR threshold on the strength of associations in ever smokers can be used to control the proportion of ‘hits’ that are not due to an effect via smoking heaviness at 5%. However, if the effect of the genetic variant on some outcomes is horizontally pleiotropic then this would inflate the FDR, such that the proportion of results below a 5% FDR P value threshold that are not due to an effect via smoking heaviness may be higher than 5%.

**Fig 3:**
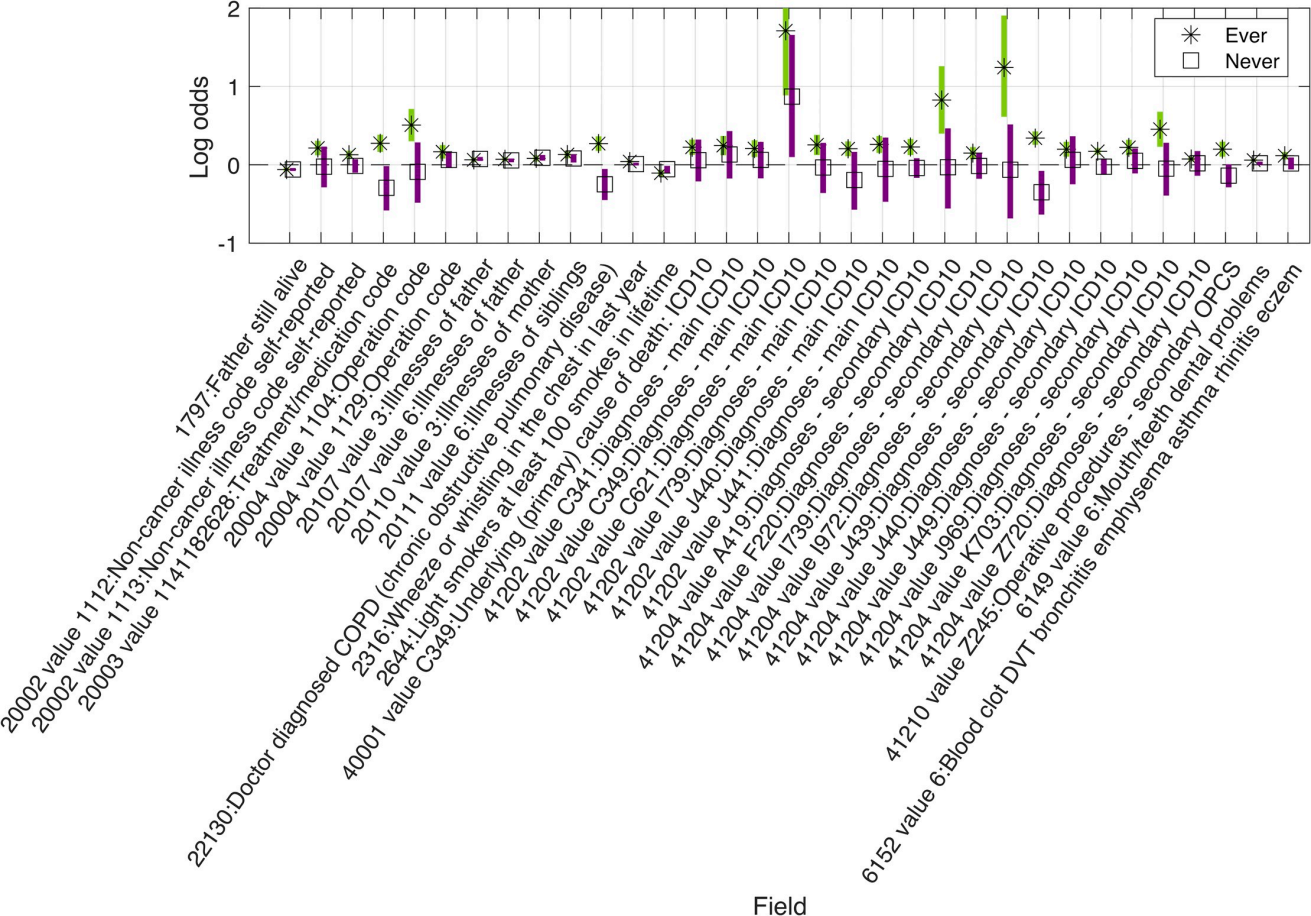
Identified main effects from MR-pheWAS in ever smokers, for binary outcomes. Results shown are those identified after correcting for multiple testing. 33 of 38 binary results are shown in this figure, with PHESANT binary result for never smokers. Results not shown in this figure are relevant to smoking participants only, e.g. ‘difficulty not smoking for 1 day’ such that they are absent in the never smokers (see S3 Table for further details).

**Fig 4:**
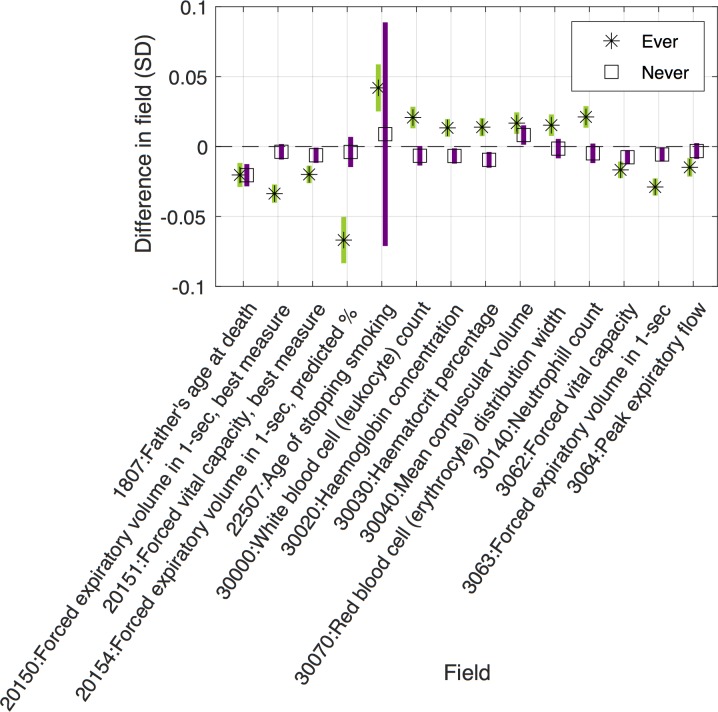
Identified main effects from MR-pheWAS in ever smokers, for continuous outcomes. Results shown are those identified after correcting for multiple testing. 14 of 18 linear results are shown in this figure, with PHESANT linear result for never smokers. Results not shown in this figure are relevant to smoking participants only, e.g. “amount of tobacco currently smoked” such that they are absent in the never smokers (see S3 Table for further details).

**Fig 5:**
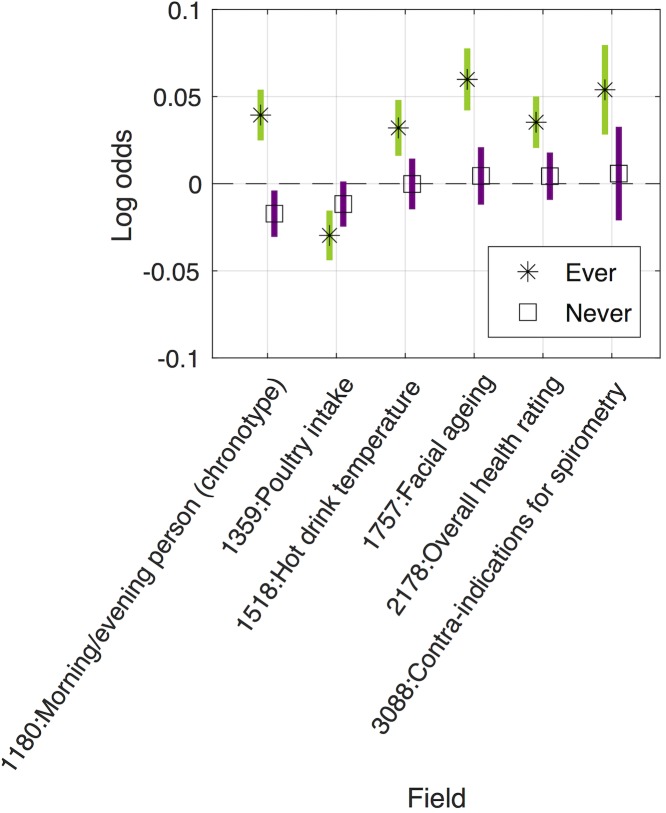
Identified main effects from MR-pheWAS in ever smokers, for ordered categorical outcomes. Results shown are those identified after correcting for multiple testing. 6 of 12 ordered categorical results are shown in this figure, with PHESANT ordered categorical result for never smokers. Results not shown in this figure are predominantly relevant to smoking participants only, e.g. “number of cigarettes smoked previously” such that they are absent in the never smokers (see S3 Table for further details).

The results of our MR-pheWAS among ever smokers includes 18 513 tests ranked by P value of the estimated effect on each outcome, given in S1 File. A QQ plot is given in Fig 2B. We identified 69 results at a false discovery rate of 5% (using a P value threshold of 0.05x69/18 513 = 1.86x10^-4^), given in Figs 3–5 and S3 Table, and of these, 32 results had a P value lower than a stringent Bonferroni corrected threshold of 2.70x10^-6^ (0.05/18 513). For comparison, we also performed MR-pheWAS among never smokers and in our full sample, and ranked these results by the strength of association. We identified 8 results at a false discovery rate of 5% among never smokers (see S4 Table), and 48 results among our full sample (see S5 Table). We also used a two-step approach to identify interactions, first ranking outcomes by the strength of association in the whole sample and then ranking the 5% FDR ‘hits’ from the first stage by the strength of interaction between ever and never smokers (P value of the Q-test statistic). This approach identified 9 results at a false discovery rate of 5% (see S6 Table).

The results identified (below the 5% FDR threshold) when ranking by our P value for interaction were a subset of those identified when ranking by the P value of the main effects among ever smokers, except for a binary outcome describing whether the participant has had a keratinizing squamous cell carcinoma (field ID [FID] = 40011, value 8071). A higher rs16969968 smoking-heaviness increasing allele dosage was associated with a higher risk of keratinizing squamous cell carcinoma among ever smokers, but a lower risk among never smokers (such that the strength of the interaction was stronger than the strength of the association in ever smokers). Overlap of results across all our MR-pheWAS is shown in S1 Fig.

#### Detailed follow-up of potentially novel results

Our identified results from our GxE MR-pheWAS (searching for interactions) included a detrimental effect of heavier smoking on facial aging (FID = 1757), where a higher genetic predisposition to heavier smoking was associated with an increased risk of looking older (as perceived by others) relative to your age. Estimates of association of rs16969968 on facial aging are shown in Table 1. Among ever smokers each additional smoking-increasing allele of rs16969968 was associated with a 1.062 [95% CI: 1.043, 1.081] higher odds of reporting an older facial aging category, and a 1.004 [95% CI: 0.988, 1.021] higher odds among never smokers (interaction P = 7.72x10^-6^). Our sensitivity analyses adjusting for age, sex and the first 40 principal components were consistent with the results of our main analyses.

**Table 1:** Results of associations of genetic instruments with facial aging outcome.

Analysis	Sample	N	Odds ratio	Interaction P value ^3^
**Associations of rs16969968 on facial aging** ^**1**^				
Main analysis	Ever smokers	137,869	1.062 [1.043, 1.081]	7.72x10^-6^
	Never smokers	167,781	1.004 [0.988, 1.021]	
Sensitivity analysis	Ever smokers	137,869	1.062 [1.043, 1.081]	7.46x10^-6^
	Never smokers	167,781	1.004 [0.988, 1.021]	
**Estimates of causal effect of lifetime smoking on facial aging** ^**2**^				
Main analysis	Ever and never smokers	305,662	1.293 [1.089, 1.534]	*NA*
Sensitivity analysis			1.294 [1.090, 1.536]	*NA*

Main analysis: Adjusted for age, sex and first 10 genetic principal components. Sensitivity Analysis: Adjusted for age, sex and first 40 genetic principal components.

^1^ Direct test of association between smoking heaviness SNP and facial aging, in ever and never smokers separately. Estimates are the change of odds of reporting looking ‘older than you are’ versus looking ‘younger than you are’ or ‘about the same’, or looking ‘older than you are’ or ‘about the same’ versus ‘younger than you are’, for each additional smoking-increasing allele of rs16969968.

^2^ Two stage IV probit regression. Estimates are the change of odds of reporting facial aging category ‘older than you are’ for a 1 SD increase in lifetime smoking score. Calculated by taking the exponent of 1.6 times the probit estimate [24].

^3^ Interaction P value generated using meta regression (metan command in Stata).

We attempted to replicate this effect using a genetic risk score for lifetime smoking exposure, a measure that incorporates duration of smoking and whether (and when) a person stopped smoking, in addition to heaviness of smoking. A 1 standard deviation (SD) higher lifetime smoking genetic instrument was associated with a 0.105 [95% confidence interval (CI): 0.102, 0.109] SD higher lifetime smoking score in our full sample, after adjusting for age, sex and the first 10 genetic principal components. We estimated that a 1SD higher lifetime smoking score caused a 1.293 [95% CI: 1.089, 1.534] higher odds of reporting that people say you look older than you are, after adjusting for age, sex and the first 10 genetic principal components.

#### Testing the impact of collider bias

Our conclusions about the causal effect of smoking heaviness on our outcomes may be affected by selection-induced collider bias [14,15]. This is because we stratified on smoking status, and we found an effect of the smoking heaviness genetic variant on smoking status. For instance, if any cause of an outcome also causes smoking status, then an association may be induced between the smoking heaviness genetic variant and that outcome (S2 Fig). If there is a true association between the genetic variant for smoking heaviness and the outcome, then it could be estimated with bias in this scenario. We performed a simulation to determine the degree of confounding needed to induce an association between the genetic variant and our identified facial aging phenotype, given there is no true causal effect of the SNP on the outcome. Further details of our simulation approach are given in Section S1 in S1 Text. In brief, we assume rs16969968 affects smoking status to the extent observed in UK Biobank and we assume a confounding variable affects both smoking status and facial aging, which creates an alternate path between the SNP and the outcome via smoking status when smoking status is conditioned on (S2 Fig). We simulate different strengths of this confounding and assess the impact on the SNP-outcome associations. Our simulation indicated that any collider bias due to the effect of the genetic variant on smoking status is likely to have had a negligible impact on the estimated effect of the genetic variant on facial aging (S3 Fig), given the same sample size and numbers of ever and never smokers as our UK Biobank sample. For example, assuming no effect of rs16969968 on the outcome, and given a confounder that is the only cause of facial aging and has a large effect on smoking status (OR = 10), the estimated effect of rs16969968 on facial aging in ever and never smokers are consistent and their confidence intervals both include the null. Hence, the degree of bias is not sufficient to (incorrectly) suggest an effect of rs16969968 on facial aging via either smoking heaviness or some other path.

The hypothesis-free nature of our analysis means that even when collider bias is small with little impact on individual estimates, across many tests it is possible that this bias inflates the false discovery rate–the proportion of ‘null’ results incorrectly identified as ‘hits’. We extended the above simulation to investigate this assuming a phenome scan restricted to continuous outcomes and found that inflation of the false discovery rate increased as the strength of the colliding relationship increased (see Supplementary section S2 for further details). For example, assuming no effect of the SNP on the outcomes, and given a confounder that has a large effect on smoking status (OR = 100) and explains 20% of the variation in the outcomes, the false discovery rate was 0.071.

## Discussion

In this study, we searched for the presence of causal effects of smoking heaviness, using the PHESANT software package to perform a GxE MR-pheWAS, by estimating the association of genetic variant rs16969968 with each outcome while restricting to ever and never smokers, respectively. We used two approaches to identify potential causal effects of smoking heaviness from our PHESANT results. Our main approach–the GxE MR-pheWAS–ranked results by the strength of interaction between ever and never smokers. This approach is commensurate with our aims, as a genetic variant that affects an outcome (at least in part) through smoking heaviness will exhibit a different effect size on this outcome, among ever and never smokers, respectively. However, identifying interactions has lower statistical power compared with identifying a main effect, especially when combined with the need to correct for the multiple tests performed in an MR-pheWAS. Our secondary approach ranked results based on the strength of the effect of rs16969968 among ever smokers and identifies causal effects under the assumption that the combined effect through smoking heaviness and any horizontal pleiotropic effect is not null. For instance, given a positive effect of a genetic variant on an outcome via smoking heaviness, and an equal but opposite effect via horizontal pleiotropy, then the effect of the variant would appear null in ever smokers and negative in never smokers, and hence this outcome would not be identified when ranking by the effect among ever smokers. Under the assumption of no horizontal pleiotropy a 5% FDR threshold would maintain the FDR of interactions at 5% as well as the FDR of effects among ever smokers. However, the proportion of results identified as a ‘hit’ among ever smokers, but for which there is no effect via smoking heaviness, increases as the prevalence of horizontal pleiotropy increases.

Our GxE MR-pheWAS of smoking heaviness (ranking on interaction strength) identified 12 results, whereas our MR-pheWAS ranking on strength of effect in ever smokers identified 69 results. Of the 12 identified in the former, 11 of these were also identified in the latter. Furthermore, the majority of results identified when ranking on the effect in ever smokers were qualitative, with an effect seen among ever smokers but not among never smokers (type E in Fig 1). Our results confirmed several established or previously reported causal effects of smoking heaviness. For example, our findings suggested higher smoking heaviness was associated with worse lung function (FIDs = {3063, 20150, 20154}) [25], higher risk of chronic obstructive pulmonary disease (FID = 22130) [25] and skin cancer (FID = 40011 value 8071) [26], and lower odds of being a morning person (FID = 1180) [27].

Our MR-pheWAS among ever smokers found only a weak negative effect of rs16969968 on BMI that did not pass the 5% FDR threshold. Previous MR studies have also identified an effect of smoking heaviness on BMI, where additional smoking-increasing alleles were associated with a lower BMI among current smokers [10,16,20]. Since we used ever smokers rather than current smokers our weaker association may be because the effect on BMI weakens over time after smoking cessation [10]. Furthermore, while previous studies found an effect of rs16969968 on BMI in never smokers [10,16,20], we found little evidence of an association in UK Biobank.

We identified other novel results, including a detrimental effect of heavier smoking on facial aging. We estimated that a 1SD increase in lifetime smoking causes a 1.29 [95% CI: 1.09, 1.53] higher odds of reporting that others say you look older than you are. A 1SD increase in lifetime smoking is, for example, equivalent to being a current smoker who has smoked 5 cigarettes per day for 12 years, or a former smoker who smoked 5 cigarettes per day for 21 years but stopped smoking 10 years ago, rather than a never smoker. This identified association should be further investigated and replicated in an independent sample, although at present we are not aware of a study with sufficient sample size and data available to do this. Our identified association may reflect a true causal effect of smoking heaviness, may be due to chance, or may arise because the genetic variants have horizontal pleiotropic effects and are thus invalid instruments for smoking heaviness or lifetime smoking. However, we examined the extent to which horizontal pleiotropy might be biasing our smoking heaviness results, by estimating the effect of the smoking heaviness genetic variant among never smokers, and found little evidence of an association, suggesting that smoking status modifies the effect of the genetic variant on facial aging. This is consistent with an effect of the genetic variant via smoking heaviness, but it is also possible that smoking status could modify a pathway from the genetic variant to facial aging that is not via smoking heaviness (see S6 Fig and discussion of limitations below). It is also possible that knowing a person’s smoking behavior might bias their perception of that person’s facial aging. However, previous research does support an effect of smoking heaviness on facial aging. For example, smoking has previously been associated with earlier facial wrinkling [28]. Furthermore, a recent study assessed perceptions of facial attractiveness using a two-alternative forced choice design where participants were randomly shown prototypical faces for smokers and non-smokers and asked to select the most attractive, and found that smoking faces were deemed less attractive [29]. Our study using MR is likely to have different sources of potential biases such that triangulation of these results adds further evidence of the detrimental effect of smoking on facial appearance in general [30,31].

Our results include examples of the “case-control by proxy” study design, where participants with relatives who are cases for a given phenotype are used as ‘proxy’ cases and those with relatives who are controls are used as ‘proxy’ controls [32]. For example, we identified associations with parental risk of smoking related diseases, such as lung cancer and emphysema, with consistent estimates in ever and never smoking subsamples, likely due to the shared genetic risk of the UK Biobank participant with their parents. Although we have not identified examples in our top results, associations may also reflect an effect of the parental genotype of UK Biobank participants on the phenotype of the UK Biobank participant. For example, maternal genotype could influence participant’s phenotype via intrauterine exposure to maternal smoking [33].

Our comparison of results across samples demonstrates the value of stratifying to detect potential causal effects. Of the 12 results identified in our GxE MR-pheWAS (testing interactions), only 5 were identified in our MR-pheWAS using the full sample. While the results of our MR-pheWAS in the full sample included associations that were not identified in our GxE MR-pheWAS in ever smokers, such as risk of operative procedures on the tarsometatarsal joint, and eye problems, these associations may be due to horizontal pleiotropy (or chance) rather than a causal effect of the smoking heaviness variant through smoking status, as these associations were consistent in ever and never smokers.

There are some limitations of this work that should be considered. First, while ranking by interaction strength between ever and never smokers is directly commensurate with our aim of identifying potential causal effects of smoking heaviness (i.e. outcomes where the effect of the genetic variant differs among ever versus never smokers), in the presence of horizontal pleiotropy this has lower statistical power compared with testing for a main effect (e.g. in ever smokers) and identified only a small number of results. Under the assumption of no horizontal pleiotropy ranking using the strength of association among ever smokers is a valid approach to identify interactions and has better statistical power. While this assumption is likely to be violated for some outcomes, in practice we identified many potentially interesting novel results with this method, including all but one of the results based on interaction strength. As GxE MR-pheWAS is a hypothesis-generating approach for discovering potentially interesting associations to be further interrogated in independent data, horizontal pleiotropy can be investigated in follow-up analyses. Follow-up is also important because we rank associations from our MR-pheWAS and GxE MR-pheWAS, such that we should expect the true strength of our strongest estimates to be less than we reported due to the winner’s curse. We also note that we test the strength of the interaction by first testing the effect of the SNP on each outcome in ever and never smokers separately and then following this up with a test for interaction. It is also possible to conduct a GxE MR-pheWAS using a model with an interaction term. This would be essentially equivalent to our stratification approach if the model also includes interactions between the genetic instrument and all covariates. However, this model approach usually assumes that the random error (i.e. variance of the error term) is the same across strata.

Second, our approach is based on the assumption that, if smoking heaviness affects an outcome then we would expect the effect of the genetic variant on the outcome to differ in ever versus never smokers, illustrated in S6A Fig. However, differing effects in smoking status strata could be because smoking status (or another factor correlated with smoking status) modifies the effect of the SNP along a pathway that is not via smoking heaviness. A hypothetical example is illustrated in S6D Fig, where the SNP affects drinking heaviness (outcome) via compulsive behaviour. In this case the effect of compulsive behaviour on drinking heaviness is modified by risky behaviour, which also affects smoking status. Thus, the effect of the SNP on drinking heaviness differs according to risky behaviour and consequently smoking status. GxE MR results should be considered in conjunction with other evidence from alternative study designs, as we have done with our facial aging result, to strengthen the evidence through triangulation [30,31]. This issue could also occur if a covariate (e.g. a genetic principal component) correlates with smoking status and interacts with the SNP, such that researchers using the GxE MR approach may wish to include interactions between each of the covariates and the SNP in their models as sensitivity analyses [34]. We also note that, while we focus on horizontal pleiotropy in this study, GxE MR can be used to assess causal effects in the presence of other violations of the exclusion restriction as long as this violation is the same across strata of the modifying factor.

Third, UK Biobank is a highly selected sample of the UK population, having a response rate of 5.5% [35]. For example, UK Biobank participants are, on average, less likely to smoke, and have less self-reported health conditions, compared with the general population [36]. Among smokers, the difference between smoking heaviness in UK Biobank compared with the general population varies by age and sex. For instance, smokers aged 45–54 years in UK Biobank, and women smokers aged 55–64 years in UK Biobank on average smoke more heavily than those in the general population, but this difference was not seen among male smokers age 55–64. Depending on the selection mechanism–i.e. how selection relates to the instrument, exposure and outcomes variables–estimates of the direct test of association of an instrumental variable on an outcome may be biased by a particular form of collider bias–selection-induced collider bias [14,15]. In general, when testing the direct association of an instrument (Z) on an outcome (Y), collider bias may occur when a third variable (C), or a descendent of C, is conditioned upon in analyses, opening up a non-causal pathway between Z and Y, for example if C is affected by Y [15]. Other scenarios (i.e. relationships of C with Z, X and Y) that may cause bias are described by Hughes et al [15]. Selection induced collider bias may occur when variable C (or a descendent of C) represents whether a person is selected into the sample (i.e. when participation in the study is conditioned upon) [15]. Hence, estimates of association between two phenotypes–such as our smoking heaviness genetic variant and a given outcome in our study–may be biased, if either smoking heaviness or the outcome or both affect selection into the study [15].

Fourth, we found an association between rs16969968 and smoking status, where each additional smoking-increasing allele of rs16969968 was associated with a 0.98 [95% CI: 0.97, 0.99] lower odds of being an ever (vs never) smoker. This means that associations may be biased by selection induced collider bias because we stratify our sample on smoking status. While our simulations indicated that any collider bias due to the SNP effect on smoking status would have a negligible impact on the estimated effect of the SNP on a given outcome, it is possible that this has increased our type 1 error rate (the proportion of null results incorrectly identified as ‘hits’) across the large number of tests performed in our MR-pheWAS. Our simulation that assessed this did show some inflation of the false discovery rate among ever smokers as the strength of the colliding relationship increased (all false discovery rates were below 0.165 in all our simulations), but this simulation included strong assumptions. For example, we assumed a particular relationship between the collider and outcomes (see S2 Fig). It is difficult to estimate the inflation experienced in our specific phenome scan where confounding structures will vary across the outcomes tested (see Supplementary section 1). We note also that selection induced collider bias may also occur if subsets of the population have already died before participating in UK Biobank or are less likely to participate due to smoking related illness. As smoking affects risk of death, it may be the case that selection induced collider bias is more extreme in the ever-smoking subsample, compared to the never-smoking subsample.

Fifth, the estimates used to identify interactions are of the direct association of the genetic variant with the outcome, and so are not estimates of the magnitude of effect of smoking heaviness. We cannot follow-up results using a formal IV analysis using these UK Biobank data to estimate a causal effect of smoking heaviness specifically because the association of rs16969968 with smoking heaviness might change across the life-course [37]. For other exposures where the association of the genetic instrument with the exposure is constant across the life-course, GxE interactions can be used to estimate the size of causal effects in the presence of horizontal pleiotropy [38].

Sixth, it is possible that reporting bias of smoking status may have biased associations. For instance, if some ‘ever’ smokers reported that they have never smoked, then for outcomes affected by rs16969968 via smoking heaviness the estimate in never smokers would be biased towards that in ever smokers. This would bias estimates of interaction between ever and never smokers towards the null. Furthermore, if the effect of smoking heaviness is transient then our interaction estimates may also be biased towards the null, because previous smokers are assigned to the ever smoker group but the effect may no longer be present. In this case, testing the interaction between current (rather than ever) versus never smokers may be the more appropriate strategy.

Seventh, our GxE MR-pheWAS used a sample of unrelated individuals such that it is possible associations may be biased due to family structure, for example, by dynastic effects [33]. Where possible, sensitivity analyses should be conducted using cohorts with data on families such that family structure can be accounted for [33].

Eighth, due to the hypothesis-free nature of a phenome scan, results generated in this way require careful consideration and follow-up. For example, our MR-pheWAS identified a potentially interesting association of rs16969968 with risk of diagnosis of the International Classification of Diseases (ICD) code ‘Descended testis’ (field 41202 value C621). This association was found in both ever and never smokers, hence we initially considered whether this may reflect an effect of parental genotype on offspring phenotype. However, further inspection revealed that this result is misleading for two reasons. First, this ICD code (C62.1) is a subcategory of ‘Malignant neoplasm of testis’ [C62]), hence pertains specifically to cancerous descended testis. Second, PHESANT deals with ICD fields by generating a binary variable for each ICD code, assigning all participants with the code as TRUE and all participants without the code as FALSE (i.e. assuming no missingness across all participants). This is not appropriate for sex specific codes, where analyses should be restricted to a particular sex. We further investigated this result by restricting to male participants, and testing the effect of rs16969968 on: 1) ‘malignant neoplasm of testis, descended’ and 2) ‘malignant neoplasm of testis, unspecified’. The latter serves as a replication for the former, under the assumption that the proportion of participants with descended versus undescended testis in the ‘unspecified’ group is the same as the ICD codes (C62.1 versus C62.0) where this is known (i.e. the majority of the unspecified group are descended; the ratio in UK Biobank is 1:16). While the positive association with the ‘descended’ group remains in both ever and never smokers (N_descended = 26; odds ratio per each additional smoking-increasing allele of rs16969968 of 3.52 [95% CI: 2.03, 6.31] in the full sample), we find little evidence of an association in the unspecified group (N_unspecified = 199; odds ratio per each additional smoking-increasing allele of rs16969968 of 0.99 [95% CI: 0.80, 1.22] in the full sample), suggesting that the association with the particular outcome is due to chance.

We used the freely available PHESANT package to search for the presence of causal effects of smoking heaviness across thousands of outcomes. While we used the rs16969968 SNP as an instrument for smoking heaviness a recent GWAS (also in UK Biobank) identified 55 smoking heaviness associated variants which could be used (e.g. combined into an allele score) in future GxE MR-pheWAS of smoking heaviness [39]. We have shown that the GxE MR-pheWAS is an effective approach to search for the presence of causal effects of an exposure using observational data, when the degree to which an exposure manifests is different across known subsets of the population. While each of the GxE MR and MR-pheWAS approaches have been used previously, this study is the first that combines these, conducting GxE MR in a hypothesis-free manner. As in hypothesis-driven GxE studies, this analysis can be used where a phenotype exists that stratifies the population into two or more groups, with different levels of the exposure. Importantly, the genetic instrument should not be associated with the phenotype used to stratify the sample, otherwise estimates may be biased due to conditioning on a collider [14]. Other potential applications include searching for the causal effects of alcohol intake (stratified by never versus ever drinkers), cannabis use (stratified by never versus ever used) and milk consumption (stratified by never versus ever drinking milk). The interaction can also be quantitative. For example, a recent GxE study assessed the effect of BMI on systolic blood pressure, using Townsend Deprivation index as the ‘interacting’ variable across which the effect of BMI was expected to vary [38]. This study also demonstrates how GxE can be used to estimate the size of causal effects in the presence of horizontal pleiotropy by first estimating the horizontal pleiotropic effect and then using this to improve the causal estimate–hence GxE MR-pheWAS can be extended to systematically (across many outcomes) estimate the size of causal effects while accounting for horizontal pleiotropy [38]. We note that estimating the magnitude of a causal effect requires stronger assumptions compared with testing for the presence of an effect, such as monotonicity of the effect of the instrument on the exposure [40]. Our approach could also be applied to qualitative gene by gene interactions. Our study of smoking heaviness serves as a model for future studies seeking to search for the causal effects of an exposure using GxE MR-pheWAS. Any potential effects identified by GxE MR-pheWAS need replication in an independent sample and assessment using other study designs with different sources of potential bias to strengthen evidence through triangulation [30].

## Materials and methods

### Study population

UK Biobank is a prospective cohort of 503 325 men and women in the UK aged between 37–73 years (99.5% were between 40 and 69 years) [41]. This cohort includes a large and diverse range of data from blood, urine and saliva samples and health and lifestyle questionnaires [42].

**Fig 6:**
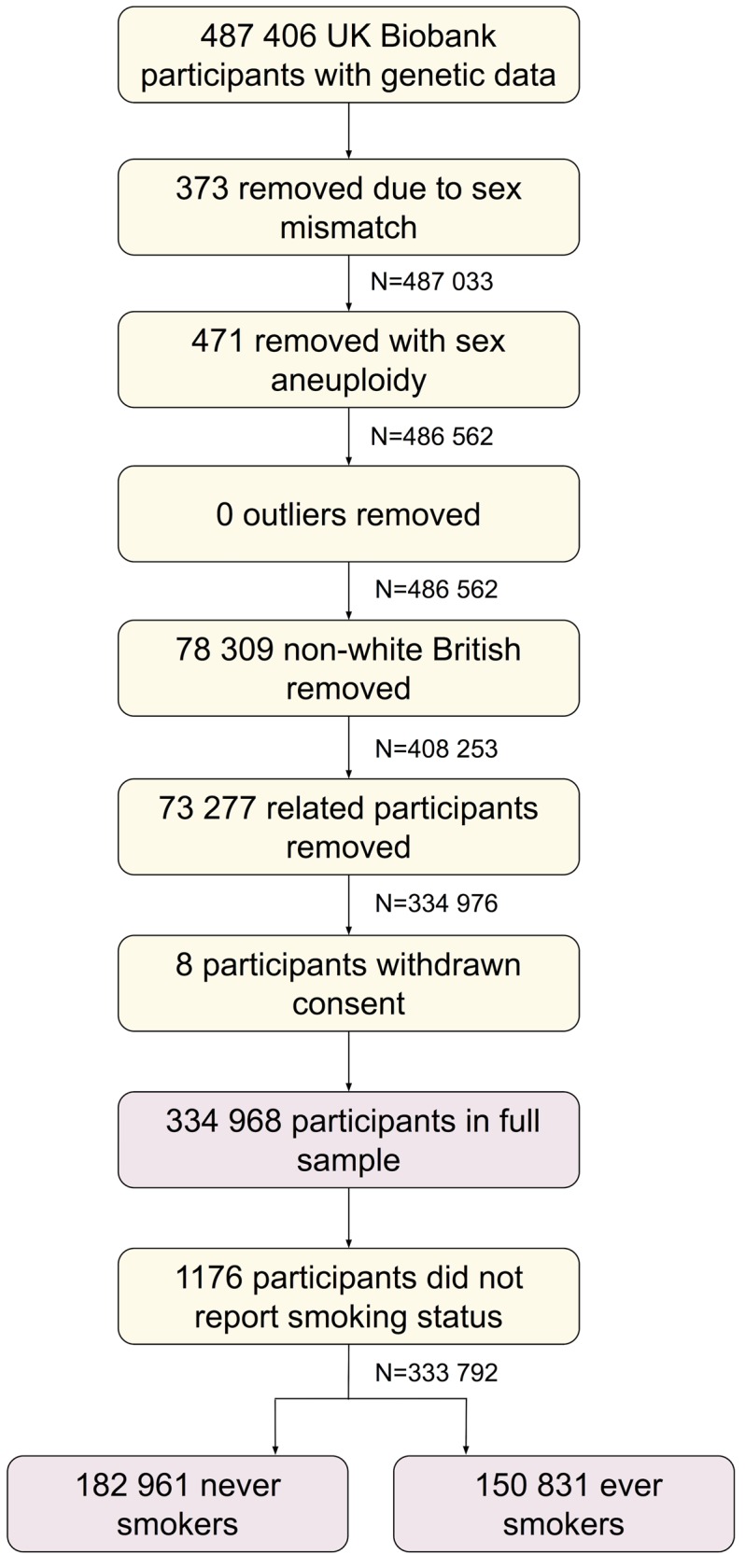
Participant flow diagram.

Of the 487 406 participants with genetic data, we removed 373 with genetic sex different to reported sex, and 471 with sex chromosome aneuploidy (identified as putatively carrying sex chromosome configurations that are not either XX or XY). We found no outliers in heterozygosity and missing rates, which would indicate poor quality of the genotypes. We removed 78 309 participants not of white British ancestry [43]. We removed 73 277 participants who were identified as being related, having a kinship coefficient denoting a third degree (or closer) relatedness [43]. We removed 8 individuals with withdrawn consent, giving a sample of 334 968 participants (we refer to as our full sample). Of these, 182 961 and 150 831 reported being never and ever (comprising previous and current) smokers, respectively. A participant flow diagram is given in Fig 6.

### Genetic variant for smoking heaviness

Two SNPs in the *CHRNA5* gene– rs16969968 and rs1051730 –that are in linkage disequilibrium (Pearson’s correlation coefficient = 0.997 in our sample), have been previously shown to be robustly associated with smoking heaviness via differences in nicotinic receptor function, and can be used interchangeably in studies of European populations [7]. We use genetic variant rs16969968 as an instrument for smoking heaviness [17], coded as the number of smoking heaviness increasing alleles.

### Outcomes

The UK Biobank data showcase allows researchers to identify variables based on the field type (http://biobank.ctsu.ox.ac.uk/showcase/list.cgi). At the time of data download there were 2761 fields of the following types: integer, continuous, categorical (single) and categorical (multiple).

We excluded 74 fields *a priori* (see S1 Table) for the following reasons: 1 field denoting the assessment centre; 2 fields described by UK Biobank as ‘polymorphic’, containing values with mixed data types; 7 fields that, although listed in the data showcase, were not currently available; 17 genetic descriptor fields, 1 sex field, 4 age fields, 17 fields describing the assessment centre environment, 4 data processing indicators, and 21 categorical (single) fields with more than one value recorded per person.

This resulted in a set of 2687 UK Biobank fields (347 integer, 1392 continuous, 836 categorical [single] and 112 categorical [multiple]), referred to hereafter as the outcome dataset (because they are tested as an outcome irrespective of whether this is biologically plausible).

### Smoking phenotypes

Smoking status was self-reported via a questionnaire at the UK Biobank assessment centre. Participants were asked to report whether they smoked previously, currently, or whether they had never smoked. We created a binary variable denoting ever versus never smokers by grouping former and current smokers.

Smoking heaviness was derived from the number of cigarettes smoked per day, which was asked via the same questionnaire, to those who reported being a previous or current smoker. We categorised the number of cigarettes into four bands: 0–10, 11–20, 21–30 and 31+.

### Covariates

We include age and sex as covariates in our models to reduce the variation in our outcomes. Age when participants attended the UK Biobank assessment centre was derived from their date of birth and the date of their assessment centre visit. Sex was self-reported during the touchscreen questionnaire (and validated using the genome-wide data). We adjusted for the first 10 genetic principal components to control for confounding via population stratification. Genetic variants are set at conception, and after conception they cannot be affected by factors that traditionally confound observational associations (such as participant socio-economic position). Also, while it is possible these factors may be on an alternative confounding pathway between the genetic variant and an outcome (e.g. via parental genotype and parental smoking heaviness [33]), it is also possible that these factors may be mediators between the participant’s genetic variant and an outcome, or a collider, such that adjusting for them would induce bias [44]. Therefore we did not adjust for any further covariates.

### Statistical methods

#### Smoking heaviness genetic variant and self-reported smoking heaviness association

We tested the association of rs16969968 with smoking heaviness using ordered logistic regression (ologit Stata command), adjusting for covariates as described above.

#### PHESANT MR-pheWAS

We searched for the causal effects of smoking heaviness, within three subsamples of UK Biobank participants: 1) ever smokers, 2) never smokers, and 3) our full sample. We conducted our MR-pheWAS in two stages. First, we ran PHESANT (version 0.17) with the ‘save’ option, to derive the PHESANT-processed outcomes for all participants in our full sample. A description of PHESANT’s automated rule-based method is given in detail elsewhere [23]. In brief, the decision rules start with the variable field type and use rules to categorize each variable as one of four data types: continuous, ordered categorical, unordered categorical or binary. Variables with the continuous and integer field type are usually assigned to the continuous data type, but some are assigned to ordered categorical if, for instance, there are only a few distinct values. Variables of the categorical (single) field type are assigned to either the binary, ordered categorical or unordered categorical, depending on whether the field has two distinct values, or has been specified as ordered or unordered in the PHESANT setup files. Variables of the categorical (multiple) field type are converted to a set of binary variables, one for each value in the categorical (multiple) fields. An inverse normal rank transform was applied to variables of the continuous data type, to ensure they were normally distributed. The derived outcomes were stored in CSV files labelled with the data type, and this information was used in the second stage.

In the second stage, for each subsample (ever, never and the full sample), we estimated the univariate association of rs16969968 with each of the outcome variables derived by PHESANT. The rs16969968 SNP and outcome are the independent (exposure) and dependent (outcome) variables in the regression model, respectively. Outcome variables with continuous, binary, ordered categorical and unordered categorical data types, were tested using linear, logistic, ordered logistic, and multinomial logistic regression, respectively. All analyses were adjusted for covariates as described above. The two-stage approach we used ensured that the same data types were assigned to each outcome across subsamples, as we process and assign data types using PHESANT on the whole sample. In each stratum, we only test outcomes with (in that stratum) at least 500 participants and at least 10 participants in each category for binary and unordered categorical variables.

#### Identifying results based on strength of interaction of ever versus never smokers

As described above, we only tested outcomes with more than 500 participants, and with at least 10 participants in each category for binary and unordered categorical variables such that the outcomes tested in each subsample may vary. For this reason, we first identified the subset of outcomes tested in both ever and never subsamples (and using the same type of regression). For this subset, we determined the strength of interaction between ever versus never smokers using meta regression (metan command in Stata), and ranked the results by the P value of the generated Q-test statistic–a measure of the heterogeneity across subgroups. We tested interactions of binary and ordered categorical results in terms of the log odds (i.e. the interactions are assumed to be multiplicative). We corrected for multiple testing by controlling for the expected proportion of false positive results. We identified the largest rank position with a P value less than *P*_*threshold*_ = 0.05×rank/n, where *n* is the total number of tests in the phenome scan. *P*_*threshold*_ is the P value threshold resulting in a false discovery rate of 5% [45]. We also calculated a highly stringent Bonferroni corrected P value threshold calculated by dividing 0.05 by the number of tests performed, which assumes each test is independent.

#### Identifying results based on strength of effect in ever smokers

If the genetic variant affects the outcome via smoking heaviness we would expect (with sufficient statistical power) to see an effect in ever smokers, except where a horizontal pleiotropic effect of the same magnitude in the opposite direction exists–these would cancel out to give a zero total effect. Hence, we conducted a secondary analysis, ranking outcomes by P value of the estimated effects of rs16969968 within the ever subsample only. To identify potential causal effects, we used both a Bonferroni and 5% FDR threshold, as described above. We examined the degree to which horizontal pleiotropy may be biasing results by viewing these estimates alongside the estimates among never smokers. We also identified top results in our never smoker and full samples, to compare the sets of results identified using each sample.

It may be reasonable to assume that rs16969968 affects most outcomes only via smoking heaviness (i.e. there is no horizontal pleiotropy), such that an association between rs16969968 and the outcome in the whole sample would indicate an effect of smoking heaviness on this outcome. We performed a two-step approach to identify interactions, similar to an approach for identifying gene-environment interactions proposed previously [46]. First, we ranked outcomes by the strength of association in the whole sample and used a 5% FDR threshold to identify results to take forward to step two. Second, we ranked these results by the strength of interaction between ever and never smokers, ranking by the P value of the Q-test statistic. This approach is valid because the strength of association in the whole sample is independent of the strength of the interaction between ever and never smokers.

#### Follow-up analyses of identified associations

We identified an association with a facial aging phenotype. Participants were asked ‘do people say you look:’ and asked to select either ‘younger than you are’, ‘about your age’ or ‘older than you are’. It is possible that the PHESANT automated approach made inappropriate decisions in its analysis, hence we re-examined this association to ensure it is not erroneous. We estimated the effect of rs16969968 on facial aging, using ordered logistic regression (ologit Stata command), among ever and never smokers respectively, adjusting for covariates as described above. We also adjusted for age, sex and the first 40 genetic principal components, as a sensitivity analysis.

We derived a measure of lifetime smoking that incorporates smoking heaviness, duration and time since cessation into a single measure [47] called the lifetime smoking index as described previously [48]. We generated a lifetime smoking genetic instrument using the 126 SNPs identified in a recent GWAS of the lifetime smoking index [48]. As this GWAS was also conducted in UK Biobank, we calculated our lifetime smoking genetic instrument as the sum of the lifetime smoking index increasing alleles (i.e. we did not weight by the effect size). We derived a binary measure of facial aging representing whether ‘people say you look older than you are’ or not, by combining the ‘younger than your age’ and ‘about your age’ categories. We determined the strength of our lifetime smoking genetic score as an instrument for lifetime smoking index using linear regression, adjusting for age, sex and the first 10 genetic principal components. We generated confidence intervals using bootstrapping (with 1000 bootstraps) as the residuals of this regression (due to non-normality of the lifetime smoking index) were not normally distributed. We estimated the causal effect of lifetime smoking on facial aging, using IV probit regression. Again, we generated confidence intervals using bootstrapping (with 1000 bootstraps) as the residuals of the first stage were not normally distributed. We took the exponent of 1.6 times the estimates, to approximate the association in terms of the change of odds [24].

Analyses are performed in R version 3.3.1 ATLAS, Matlab r2018a or Stata version 15, and code is available at [https://github.com/MRCIEU/PHESANT-MR-pheWAS-smoking]. Git tag v0.4 corresponds to the version presented here.

## Supporting information

S1 Text Simulating collider bias, methods and results. (PDF)

S1 Table UK Biobank fields excluded from smoking heaviness MR-pheWAS. (PDF)

S2 Table Results from smoking heaviness GxE MR-pheWAS below P values threshold of 3.59x10^-5^ with a false discovery rate of 5%, ranked by interaction P value. Total number of tests with ever and never result, and same regression type = 16692. Bonferroni threshold = 0.05/16692 = 3.00x10^-6^. False discovery rate threshold = 0.05x12/16692 = 3.59x10^-5^. ^1^ Linear: associations are the SD difference in inverse rank normal transformed outcome for each additional smoking-increasing allele of rs16969968. Ordered: Associations are the log odds of a higher outcome group for each additional smoking-increasing allele of rs16969968. Binary: Associations are the log odds of comparison versus baseline outcome group for each additional smoking-increasing allele of rs16969968. ^2^ Strength of interaction between ever versus never smokers using meta regression (metan command in Stata). ^3^ Information on categories for multinomial, ordinal and binary regression results: reference category for multinomial regression results, baseline category for binary logistic regression results, and category ordering for ordered logistic regression results. For example, *{Younger than you are … Older than you are}*” for field 1757 means that there are categories ranging from “*Younger than you are”* to “*Older than you are”* where the former is coded with the smallest value, and the latter with the largest value. ^4^ In addition to the field ID, this column also contains the reference value for multinomial regression results, and the field value for which a binary variable was generated for categorical (multiple) fields.
(PDF)

S3 Table Results from smoking heaviness MR-pheWAS below P values threshold of 1.86x10^-4^ with a false discovery rate of 5%, ranked by P value among ever smokers. Results of main analysis, adjusting for age, sex and the first 10 genetic principal components. ^1^ Direction of change of outcome with genetic predisposition to higher smoking heaviness. ^2^ For multinomial logistic regression results a single P value was calculated for each model as a whole, using the likelihood ratio chi-square test. ^3^ Information on categories for multinomial, ordinal and binary regression results: reference category for multinomial regression results, baseline category for binary logistic regression results, and category ordering for ordered logistic regression results. For example, “*{thinner … plumper}*” for field 1687 means that there are categories ranging from thinner to plumper where thinner is coded with the smallest value and plumper with the largest value. ^4^ In addition to the field ID, this column also contains the reference value for multinomial regression results, and the field value for which a binary variable was generated for categorical (multiple) fields. ^5^ Where test type differs in never smokers this is shown in brackets. Bonferroni threshold = 2.70x10^-6^ (0.05/18513); false discovery rate threshold = 0.05x69/18513 = 1.86x10^-4^. Binary, linear and ordered results in this table are shown in Figs 3–5 in main paper (one result was an unordered categorical result [Field 1448 reference 3], and is not shown as P value was generated using a likelihood ratio test such that an estimate and confidence interval is not available).
(PDF)

S4 Table Results from smoking heaviness MR-pheWAS below P values threshold of 2.23x10^-5^ with a false discovery rate of 5%, ranked by P value among never smokers. Total number of tests in never smokers = 17975. Bonferroni threshold = 0.05/17975 = 2.78x10^-6^. False discovery rate threshold = 0.05x8/17975 = 2.23x10^-5^.
(PDF)

S5 Table Results from smoking heaviness MR-pheWAS below P values threshold of 1.04x10^-4^ with a false discovery rate of 5%, ranked by P value among all participants. Total number of tests in whole sample = 23009. Bonferroni threshold = 0.05/23009 = 2.17x10^-6^. False discovery rate threshold = 0.05x48/23009 = 1.04x10^-4^.
(PDF)

S6 Table Results from smoking heaviness MR-pheWAS using two-step approach. Step 1: Rank results by association strength among whole sample, identifies 48 results (S4 Table), of which 36 have an interaction P value for use in step 2. Step 2: Rank results by strength of interaction of ever versus never smokers. Bonferroni threshold = 0.05/36 = 1.39x10^-3^. False discovery rate threshold = 0.05x9/36 = 1.25x10^-2^. ^1^ Linear: associations are the SD difference in inverse rank normal transformed outcome for each additional smoking-increasing allele of rs16969968. Ordered: Associations are the log odds of a higher outcome group for each additional smoking-increasing allele of rs16969968. Binary: Associations are the log odds of comparison versus baseline outcome group for each additional smoking-increasing allele of rs16969968. ^2^ Strength of interaction between ever versus never smokers using meta regression (metan command in Stata). ^3^ Information on categories for multinomial, ordinal and binary regression results: reference category for multinomial regression results, baseline category for binary logistic regression results, and category ordering for ordered logistic regression results. For example, *{Younger than you are … Older than you are}*” for field 1757 means that there are categories ranging from “*Younger than you are”* to “*Older than you are”* where the former is coded with the smallest value, and the latter with the largest value. ^4^ In addition to the field ID, this column also contains the reference value for multinomial regression results, and the field value for which a binary variable was generated for categorical (multiple) fields.
(PDF)

S1 Fig Overlap of ‘top’ results across our MR-pheWAS analyses in ever smokers, never smokers and the full sample, and our GxE MR-pheWAS ranking based on interaction strength. (PDF)

S2 Fig Illustration of simulated collider bias. In our UK Biobank sample the smoking heaviness genetic variant is estimated to affect both smoking status and the identified outcome phenotypes. If a confounder exists that affects both smoking status and a given outcome then smoking status is a collider and stratifying on smoking status may cause collider bias–bias in the estimated effect of the genetic variant on the outcome phenotype. An exception to this is when the genetic variant for smoking heaviness does not affect the outcome, and they act additively on smoking status (i.e. there is no additive interaction on the log-probability scale). In this case, collider bias would not occur.
(PDF)

S3 Fig Results of collider bias simulations of facial aging, with range of effect sizes of confounder on outcome. OR_conf,si_ is the odds ratio of the confounder on smoking status, i.e. the change of odds of being an ever versus never smoker for a 1 standard deviation increase in confounder. r2 is proportion of the variance of the continuous facial aging phenotype (underlying the categorical facial aging outcome) that is explained by the confounder. a-d: positive effect of confounder on outcome, with OR of the confounder on smoking status of 10 (a), 20 (b), 50 (c) and 100 (d). e-h: negative effect of confounder on outcome, with OR of the confounder on smoking status of 10 (e), 20 (f), 50 (g) and 100 (h).
(PDF)

S4 Fig Results of collider bias simulation of continuous outcome, with range of effect sizes of confounder on outcome. OR_conf,si_ is the odds ratio of the confounder on smoking status, i.e. that change of odds of being an ever versus never smoker for a 1 standard deviation increase in confounder. r2 is proportion of the variance of the continuous phenotype that is explained by the confounder. a-d: positive effect of confounder on outcome, with OR of the confounder on smoking status of 10 (a), 20 (b), 50 (c) and 100 (d). e-h: negative effect of confounder on outcome, with OR of the confounder on smoking status of 10 (e), 20 (f), 50 (g) and 100 (h).
(PDF)

S5 Fig Illustration of bias when SNP has a greater effect on smoking status compared with the effect seen in UK Biobank. These simulations assume a large (and hence unlikely) effect of the confounder on smoking status (odds ratio = 100; i.e. we are assuming a 1 standard deviation change of the confounder causes a 100 higher odds of being an ever smoker). Furthermore, we assume a 1 dosage increase in SNP causes a 0.8 lower odds of being an ever smoker (i.e. we have made this more extreme than we see in UK Biobank for illustration purposes). (a) Categorical facial aging outcome, positive effect of confounder on outcome. (b) Continuous outcome, positive effect of confounder on outcome. (c) Categorical facial aging outcome, negative effect of confounder on outcome. (d) Continuous outcome, negative effect of confounder on outcome. In plots (a) and (c) r2 is proportion of the variance of the continuous facial aging phenotype (underlying the categorical facial aging outcome) that is explained by the confounder. In plots (b) and (d) r2 is proportion of the variance of the continuous phenotype that is explained by the confounder.
(PDF)

S6 Fig Illustration of pathways that do and do not cause an interaction due to smoking status. Solid black boxes indicate an interaction. Box around smoking status variable indicates that this variable is conditioned upon (i.e. we stratify on ever versus never smokers). Please see code in the project’s GitHub repository [http://github.com/MRCIEU/PHESANT-MR-pheWAS-smoking/] for simulations of these scenarios. a) Our hypothesised pathway, where the genetic variant affects the outcome via smoking heaviness. b) Independent effects of the SNP and smoking status on the outcome are not problematic–the effect estimates in smoking status strata (ever versus never) are still consistent. c) If there is only an effect on the outcome via smoking status, rather than smoking heaviness, then within smoking status strata there is no association between the SNP and the outcome. d) An interaction of smoking status along the pathway between the SNP and outcome would also give an interaction of the effects of the SNP across smoking status strata.
(PDF)

S7 Fig Example violation of exclusion restriction assumption that is not horizontal pleiotropy, but for which GxE MR can be used to identify causal effects. In this figure we show two directed acyclic graphs (DAGs), after stratification by ever versus never smokers (of the UK Biobank participants). We assume an effect of parental smoking heaviness on the UK Biobank participants outcome. This alternative pathway between the UK Biobank participant's SNP and the outcome (via parental SNP and parental smoking heaviness) is the same in UK Biobank ever and never smokers. It is not horizontal pleiotropy because there is no causal effect of the SNP (or more precisely the genetic variation for which the SNP is tagging) on the outcome–it is the parental genetic variation that affects the outcome. Note also that this assumes participant smoking status is independent of parental smoking status, otherwise the extent to which this alternative pathway manifests would vary in UK Biobank participant ever versus never smokers.
(PDF)

S1 File Results from smoking heaviness MR-pheWAS. (TSV)
