# Supplementary material for: MR-pheWAS with stratification and interaction: Searching for the causal effects of smoking heaviness identified an effect on facial aging
Source: PLoS Genet. 2019 Oct 31;15(10):e1008353. doi: 10.1371/journal.pgen.1008353 (PMC6822717; doi:10.1371/journal.pgen.1008353)
Supplement: S1 Text — (PDF) [file pgen.1008353.s001.pdf]

MR-pheWAS with stratification and interaction:
Searching for the causal effects of smoking heaviness identified an effect on facial aging

SUPPLEMENTARY INFORMATION

S1 text: Simulating collider bias

We perform a simulation to determine the degree of confounding that would cause collider bias, i.e. induce an association between the smoking heaviness SNP and an outcome, given that there is no true causal effect of the SNP on the outcome. In this simulation collider bias is induced due to a confounding factor that affects both the outcome and smoking initiation (see S2 fig). All continuous variables are standardised.

To perform this simulation, we first generate the SNP variable according to the allelic dosage distribution in UK Biobank. We model two independent effects of the confounding factor and the SNP, on smoking status. The probability of smoking initiation for each participant, due to the SNP and confounder, is generated using a logistic model. The SNP parameter is estimated empirically from our UK Biobank sample. We repeat our simulation, each time assuming a different effect of the confounder on smoking status, with odds ratios 10, 20, 50, and 100 (i.e. we are assuming a 1 standard deviation change of the confounder causes a [10,20,50 or 100] higher odds of being an ever smoker). We set the intercept term so that the proportion of ever smokers is consistent with the proportion in UK Biobank (45%). We sample according to these probabilities such that each participant is assigned a binary value, denoting whether they are an ever smoker.

Given an effect of the SNP on smoking status, and an effect of the confounder on the outcome, collider bias may occur if there is also an effect of the confounding factor on smoking status (see again S2 fig.). To determine whether our facial aging finding may be due to collider bias, we generate a continuous facial aging variable that is a function of a random variable and the confounder (with a sample size of 305650 as in our UK Biobank facial aging analysis) – i.e. we assume no true association between our genetic instrument and facial aging. We perform our simulations twice, with a positive and negative effect of the confounder on facial aging, respectively. We then generate a categorical facial aging variable by splitting this continuous facial aging variable into three strata, with the same proportions in each category as the facial aging phenotype in UK Biobank.

We incrementally change the proportion of variance of the continuous facial aging outcome that is explained by the confounder, to identify the degree of confounding needed to induce collider bias between the SNP and facial aging (i.e. associations between the SNP and facial aging within smoking status strata). Simulation code can be found in the GitHub repository [https://github.com/MRCIEU/PHESANT-MR-pheWAS-smoking].

The results of our simulations are shown in S3 fig. We did not see estimated effects in ever smokers as strong as we identified for facial aging (odds ratio [OR] 1.062 [95% CI: 1.043, 1.081]). This suggests that collider bias is likely to have had a negligible impact on our facial aging finding.

S4 fig. shows the results of these simulations using the continuous facial aging phenotype instead of the categorical version, to demonstrate whether collider bias is likely to be biasing associations of continuous outcomes. These simulations suggest that collider bias is likely to be negligible for continuous outcomes. These show that collider bias is likely to be neglibible for continuous outcomes in our MR-pheWAS because the association between the SNP and smoking status is weak.

For simulations using both the categorical and continuous facial aging phenotypes we found that collider bias is likely to have a negligible impact on our results, and this is because the effect of the SNP on smoking status is small. To demonstrate this, we repeated our simulation assuming a stronger effect of the SNP on smoking status (OR is set to 0.8, rather than 0.98). S5 fig. shows that an association between the SNP and outcome (continuous or categorical) is induced in this instance. Furthermore, the SNP effect on the categorical facial aging phenotype interacts with smoking status.

Effect on the false discovery rate in phenome scans

While the collider bias has little effect on the estimates for individual phenotypes, it is possible that across the very large number of outcomes tested in our phenome scan this collider bias could cause an inflation on the proportion of 'null' results incorrectly identified as 'hits' (the false discovery rate [FDR]). We investigated this by extending our simulations described above for the continuous facial aging phenotype. We assumed an odds ratio of 100 for the effect of the confounder on smoking initiation, as an extreme example. As above, we incrementally changed the proportion of variance of the continuous facial aging outcome that is explained by the confounder, but for each of these we now generate 1000 simulated outcomes. We calculate the false discovery rate as the number of results with a P value < 0.05.

We found 48, 71, 92, 89, 144 and 165 of the 1000 tests were associated with P value < 0.05 for the simulations where the proportion of variance of the continuous facial aging outcome that is explained by the confounder was 0, 0.2, 0.4, 0.6, 0.8 and 1, respectively (i.e. the FDRs were 0.048, 0.071, 0.092, 0.089, 0.144 and 0.165). Thus, as expected when there is no collider bias, because the confounder (between smoking status and the outcome) does not affect the outcome such that this path via smoking status is broken, the FDR is around 0.05. As the strength of the effect of the confounder on the outcome increases, so does the collider bias and this inflates the FDR. In this simulation we have made various assumptions (e.g. the confounding structures through which smoking status and the outcome are related) that affect the degree of FDR inflation such that we cannot determine the true extent of FDR inflation in our phenome scan.
