# Supplementary material for: MR-pheWAS with stratification and interaction: Searching for the causal effects of smoking heaviness identified an effect on facial aging
Source: PLoS Genet. 2019 Oct 31;15(10):e1008353. doi: 10.1371/journal.pgen.1008353 (PMC6822717; doi:10.1371/journal.pgen.1008353)
Supplement: S1 Table — (PDF) [file pgen.1008353.s002.pdf]

**Number of fields**	**Field IDs**	**Reason excluded from phenome scan**
1	54	Assessment centre
2	87, 92	Polymorphic fields (containing values with mixed data types)
7	5990, 22014, 22015, 22050, 23207, 23211, 23294, 23303, 24025, 110003	Not available at time of data download
17	22000, 22001, 22003, 22004, 22005, 22006, 22009, 22010, 22011, 22012, 22013, 22018, 22019, 22021, 22027, 22051, 22052	Genetic data description fields
1	31	Sex field
4	34, 52, 21003, 21022	Age fields
17	20012, 20013, 20014, 3059, 3065, 3081, 4268, 4275, 4281, 4287, 5149, 5152, 5155, 5164, 6024, 6074, 6075	Assessment centre environment (ACE) fields
4	22411, 22412, 22413, 22414	Data processing indicators
21	4232, 4243, 4259, 5090, 5091, 5136, 5138, 5139, 5140, 5141, 5142, 5143, 5144, 5145, 5146, 5147, 5148, 6312, 401, 402, 10691	Categorical (single) field with more than one value recorded per person.
