# Supplementary material for: MR-pheWAS with stratification and interaction: Searching for the causal effects of smoking heaviness identified an effect on facial aging
Source: PLoS Genet. 2019 Oct 31;15(10):e1008353. doi: 10.1371/journal.pgen.1008353 (PMC6822717; doi:10.1371/journal.pgen.1008353)
Supplement: S2 Table — Total number of tests with ever and never result, and same regression type = 16692. Bonferroni threshold = 0.05/16692 = 3.00x10^-6^. False discovery rate threshold = 0.05x12/16692 = 3.59x10^-5^. 1 Linear: associations are the SD difference in inverse rank normal transformed outcome for each additional smoking-increasing allele of rs16969968. Ordered: Associations are the log odds of a higher outcome group for each additional smoking-increasing allele of rs16969968. Binary: Associations are the log odds of comparison versus baseline outcome group for each additional smoking-increasing allele of rs16969968. 2 Strength of interaction between ever versus never smokers using meta regression (metan command in Stata). 3 Information on categories for multinomial, ordinal and binary regression results: reference category for multinomial regression results, baseline category for binary logistic regression results, and category ordering for ordered logistic regression results. For example, {Younger than you are … Older than you are}” for field 1757 means that there are categories ranging from “Younger than you are” to “Older than you are” where the former is coded with the smallest value, and the latter with the largest value. 4 In addition to the field ID, this column also contains the reference value for multinomial regression results, and the field value for which a binary variable was generated for categorical (multiple) fields. (PDF) [file pgen.1008353.s003.pdf]

Rank	Field ID [4]	Interaction P value [2]	Association among ever smokers [1]	Association among never smokers [1]	Regression Type	Field description	Reference, baseline or ordinal categories [3]
1	20150	7.61e-12	-0.034 [-0.040, -0.027]	-0.004 [-0.009, 0.002]	LINEAR	Forced expiratory volume in 1-second (FEV1), Best measure	-
2	20154	4.49e-10	-0.067 [-0.083, -0.050]	-0.003 [-0.015, 0.007]	LINEAR	Forced expiratory volume in 1-second (FEV1), predicted percentage	-
3	3063	1.06e-08	-0.029 [-0.035, -0.023]	-0.006 [-0.011, -0.001]	LINEAR	Forced expiratory volume in 1-second (FEV1)	-
4	1180	1.92e-08	0.039 [0.025, 0.054]	-0.017 [-0.031, -0.004]	ORDERED	Morning/evening person (chronotype)	{Definitely a 'morning' person … Definitely an 'evening' person}
5	30030	8.52e-08	0.014 [0.007, 0.020]	-0.009 [-0.151, 0.004]	LINEAR	Haematocrit percentage	-
6	30000	2.60e-07	0.021 [0.013, 0.028]	-0.007 [-0.014, 0.000]	LINEAR	White blood cell (leukocyte) count	-
7	30140	9.67e-07	0.021 [0.013, 0.029]	-0.005 [-0.012, 0.002]	LINEAR	Neutrophill count	-
8	30020	2.29e-06	0.013 [0.007, 0.020]	-0.007 [-0.012, -0.001]	LINEAR	Haemoglobin concentration	-
Bonferroni threshold							
9	22130	3.52e-06	0.271 [0.178, 0.364]	-0.248 [-0.450, -0.052]	BINARY	Doctor diagnosed COPD (chronic obstructive pulmonary disease)	No
10	41204 value J439	3.54e-06	0.342 [0.256, 0.427]	-0.350 [-0.635, -0.077]	BINARY	Diagnoses - secondary ICD10: Emphysema, unspecified	No
11	40011 value 8071	5.70e-06	0.369 [0.151,0.584]	-0.558 [-0.904, -0.232]	BINARY	Histology of cancer tumour: Squamous cell carcinoma, keratinizing	No
12	1757	7.71e-06	0.060 [0.042,0.078]	0.004 [-0.012, 0.021]	ORDERED	Facial ageing	*{Younger than you are … Older than you are}*
