# Supplementary material for: MR-pheWAS with stratification and interaction: Searching for the causal effects of smoking heaviness identified an effect on facial aging
Source: PLoS Genet. 2019 Oct 31;15(10):e1008353. doi: 10.1371/journal.pgen.1008353 (PMC6822717; doi:10.1371/journal.pgen.1008353)
Supplement: S3 Table — Results of main analysis, adjusting for age, sex and the first 10 genetic principal components. 1 Direction of change of outcome with genetic predisposition to higher smoking heaviness. 2 For multinomial logistic regression results a single P value was calculated for each model as a whole, using the likelihood ratio chi-square test. 3 Information on categories for multinomial, ordinal and binary regression results: reference category for multinomial regression results, baseline category for binary logistic regression results, and category ordering for ordered logistic regression results. For example, “{thinner … plumper}” for field 1687 means that there are categories ranging from thinner to plumper where thinner is coded with the smallest value and plumper with the largest value. 4 In addition to the field ID, this column also contains the reference value for multinomial regression results, and the field value for which a binary variable was generated for categorical (multiple) fields. 5 Where test type differs in never smokers this is shown in brackets. Bonferroni threshold = 2.70x10^-6^ (0.05/18513); false discovery rate threshold = 0.05x69/18513 = 1.86x10^-4^. Binary, linear and ordered results in this table are shown in Figs 3–5 in main paper (one result was an unordered categorical result [Field 1448 reference 3], and is not shown as P value was generated using a likelihood ratio test such that an estimate and confidence interval is not available). (PDF) [file pgen.1008353.s004.pdf]

Rank	Field ID [4]	P value [2]	PHESANT Association in ever smokers [1]	PHESANT Association in never smokers [1]	Regression Type in ever smokers [5]	Field description	Reference, baseline or ordinal categories [3]
1	2887	1.08e-73	0.194 [0.173, 0.215]	*EVER ONLY PHENO*	ORDERED	Number of cigarettes previously smoked daily	*{3 quantity bands}*
2	20162	4.13e-73	0.084 [0.075, 0.093]	*EVER ONLY PHENO*	LINEAR	Pack years adult smoking as proportion of life span exposed to smoking	-
3	20161	4.04e-70	0.081 [0.072, 0.090]	*EVER ONLY PHENO*	LINEAR	Pack years of smoking	-
4	1249	8.00e-47	-0.130 [-0.147, -0.112]	0.019 [0.004,0.035]	ORDERED [BINARY]	Past tobacco smoking	*{Smoked on most or all days … I have never smoked}*
5	3456	1.26e-27	0.206 [0.169, 0.244]	*EVER ONLY PHENO*	ORDERED	Number of cigarettes currently smoked daily (current cigarette smokers)	*{3 quantity bands}*
6	20150	6.65e-24	-0.034 [-0.040, -0.027]	-0.004 –0.009, 0.002]	LINEAR	Forced expiratory volume in 1-second (FEV1), Best measure	-
7	3476	1.40e-22	0.180 [0.144, 0.216]	*EVER ONLY PHENO*	ORDERED	Difficulty not smoking for 1 day	{Very easy … Very difficult}
8	3063	2.02e-20	-0.029 [-0.035, -0.023]	-0.006 [-0.011, -0.001]	LINEAR	Forced expiratory volume in 1-second (FEV1)	-
9	20154	2.91e-15	-0.067 [-0.083, -0.050]	-0.004 [-0.015, 0.007]	LINEAR	Forced expiratory volume in 1-second (FEV1), predicted percentage	-
10	41204 value J449	4.46e-15	0.172 [0.129, 0.216]	-0.023 [-0.129, 0.080]	BINARY	Diagnoses - secondary ICD10: Chronic obstructive pulmonary disease, unspecified	No
11	41204 value J439	5.62e-15	0.342 [0.256, 0.427]	-0.350 [-0.635, -0.077]	BINARY	Diagnoses - secondary ICD10: Emphysema, unspecified	No
12	3466	4.03e-13	-0.131 [-0.167, -0.096]	*EVER ONLY PHENO*	ORDERED	Time from waking to first cigarette	*{Less than 5 minutes … Longer than 2 hours}*
13	1757	4.30e-11	0.060 [0.042, 0.078]	0.004 [-0.012, 0.021]	ORDERED	Facial ageing	*{Younger than you are … older than you are}*
14	20151	4.74e-10	-0.020 [-0.026, -0.014]	-0.006 [-0.012, -0.001]	LINEAR	Forced vital capacity (FVC), Best measure	-
15	6149 value 6	7.01e-10	0.060 [0.041, 0.079]	0.020 [-0.002, 0.042]	BINARY	Mouth/teeth dental problems: Dentures	No
16	22130	1.08e-08	0.271 [0.178, 0.364]	-0.248 [-0.450, -0.052]	BINARY	Doctor diagnosed COPD (chronic obstructive pulmonary disease)	No
17	20107 value 6	1.62e-08	0.071 [0.047, 0.096]	0.057 [0.033, 0.080]	BINARY	Illnesses of father: Chronic bronchitis/emphysema	No
18	3062	3.30e-08	-0.017 [-0.023, -0.011]	-0.008 [-0.013, -0.003]	LINEAR	Forced vital capacity (FVC)	-
19	20111 value 6	5.28e-08	0.131 [0.084, 0.178]	0.081 [0.029, 0.133]	BINARY	Illnesses of siblings: Chronic bronchitis/emphysema	No
20	30140	7.39e-08	0.021 [0.013, 0.029]	*-0.005 [-0.012, 0.002]*	LINEAR	Neutrophill count	-
21	2926	1.02e-07	0.054 [0.034, 0.074]	*EVER ONLY PHENO*	ORDERED	Number of unsuccessful stop-smoking attempts	*{3 quantity bands}*
22	1180	1.14e-07	0.039 [0.025, 0.054]	-0.017 [-0.031, -0.004]	ORDERED	Morning/evening person (chronotype)	{Definitely a 'morning' person … Definitely an 'evening' person}
23	30000	1.31e-07	0.021 [0.013, 0.028]	-0.007 [-0.014, 0.000]	LINEAR	White blood cell (leukocyte) count	-
24	1797	3.35e-07	-0.060 [-0.082, -0.037]	-0.059 [-0.078, -0.040]	BINARY	Father still alive	No
25	2644	8.58e-07	-0.105 [-0.147, -0.063]	-0.056 [-0.099, -0.012]	BINARY	Light smokers, at least 100 smokes in lifetime	No
26	6157 value 1	9.66e-07	0.079 [0.048, 0.111]	*EVER ONLY PHENO*	BINARY	Why stopped smoking: Illness or ill health	No
27	22507	1.03e-06	0.042 [0.025, 0.059]	0.009 [-0.071, 0.089]	LINEAR	Age of stopping smoking	-
28	20003 value 1141182628	1.05e-06	0.275 [0.164, 0.385]	-0.293 [-0.582, -0.015]	BINARY	Treatment/medication code: tiotropium	No
29	20002 value 1113	1.20e-06	0.129 [0.077, 0.181]	-0.014 [-0.101, 0.072]	BINARY	Non-cancer illness code, self-reported: emphysema/chronic bronchitis	No
30	20004 value 1104	1.40e-06	0.508 [0.300, 0.713]	-0.089 [-0.484, 0.286]	BINARY	Operation code: aortic aneurysm/repair or stent	No
31	6152 value 6	1.50e-06	0.117 [0.069, 0.164]	0.018 [-0.059, 0.094]	BINARY	Blood clot, DVT, bronchitis, emphysema, asthma, rhinitis, eczema, allergy diagnosed by doctor: Emphysema/chronic bronchitis	No

32	41202 value J441	2.39e-06	0.260 [0.152, 0.368]	-0.052 [-0.471, 0.348]	BINARY	Diagnoses - main ICD10: Chronic obstructive pulmonary disease with acute exacerbation, unspecified	No
Bonferroni threshold							
33	2178	2.89e-06	0.035 [0.021, 0.050]	0.004 [-0.009, 0.018]	ORDERED	Overall health rating	{Excellent … poor}
34	2316	3.61e-06	0.042 [0.024, 0.059]	0.010 [-0.009, 0.029]	BINARY	Wheeze or whistling in the chest in last year	No
35	1807	3.75e-06	-0.020 [-0.029, -0.012]	-0.020 [-0.028, -0.013]	LINEAR	Father's age at death	-
36	41204 value Z720	5.07e-06	0.074 [0.042, 0.106]	0.020 [-0.140, 0.176]	BINARY	Diagnoses - secondary ICD10: tobacco use	No
37	20002 value 1112	5.11e-06	0.215 [0.122, 0.307]	-0.023 [-0.287, 0.233]	BINARY	Non-cancer illness code, self-reported: chronic obstructive airways disease/COPD	No
38	20107 value 3	5.48e-06	0.064 [0.036, 0.091]	0.075 [0.050, 0.100]	BINARY	Illnesses of father: Lung cancer	No
39	40001 value C349	7.07e-06	0.225 [0.126, 0.323]	0.059 [-0.212, 0.323]	BINARY	Underlying (primary) cause of death: ICD10: Malignant neoplasm of bronchus and lung, unspecified	No
40	41204 value J969	8.02e-06	0.222 [0.124, 0.319]	0.051 [-0.110, 0.210]	BINARY	Diagnoses - secondary ICD10: Respiratory failure, unspecified	No
41	22508	8.03e-06	0.165 [0.093, 0.237]	*EVER ONLY PHENO*	LINEAR	Amount of tobacco currently smoked	-
42	3064	1.21e-05	-0.015 [-0.022, -0.008]	-0.003 [-0.009, 0.002]	LINEAR	Peak expiratory flow (PEF)	-
43	41204 value A419	1.37e-05	0.228 [0.125, 0.330]	-0.040 [-0.167, 0.085]	BINARY	Diagnoses - secondary ICD10: Septicaemia, unspecified	No
44	2907	1.76e-05	-0.046 [-0.067, -0.025]	*EVER ONLY PHENO*	BINARY	Ever stopped smoking for 6+ months	No
45	30040	1.89e-05	0.017 [0.009, 0.024]	0.008 [0.001, 0.015]	LINEAR	Mean corpuscular volume	-
46	41202 value J440	2.38e-05	0.205 [0.110, 0.300]	-0.194 [-0.573, 0.166]	BINARY	Diagnoses - main ICD10: Chronic obstructive pulmonary disease with acute lower respiratory infection	No
47	30030	2.41e-05	0.014 [0.007, 0.020]	-0.009 [-0.015, -0.004]	LINEAR	Haematocrit percentage	-
48	30020	3.16e-5	0.013 [0.007, 0.020]	-0.007 [-0.012, -0.001]	LINEAR	Haemoglobin concentration	-
49	3088	3.94e-05	0.054 [0.028, 0.080]	0.006 [-0.021, 0.033]	ORDERED	Contra-indications for spirometry	{No … Yes}
50	41204 value J440	4.25e-05	0.200 [0.104, 0.295]	0.064 [-0.248, 0.365]	BINARY	Diagnoses - secondary ICD10: Chronic obstructive pulmonary disease with acute lower respiratory infection	No
51	1359	4.47e-05	-0.030 [-0.044, -0.015]	-0.012 [-0.025, 0.001]	ORDERED	Poultry intake	{Never … Once or more daily}
52	20110 value 3	4.88e-05	0.082 [0.043, 0.122]	0.090 [0.054, 0.126]	BINARY	Illnesses of mother: Lung cancer	No
53	41210 value Z245	4.89e-05	0.200 [0.103, 0.296]	-0.138 [-0.287, 0.008]	BINARY	Operative procedures - secondary OPCS: Bronchus	No
54	1448 (reference 3)	6.44e-05	-	-	MULTINOMIAL	Bread type	Wholemeal or wholegrain
55	41204 value K703	6.87e-05	0.455 [0.230, 0.678]	-0.049 [-0.393, 0.281]	BINARY	Diagnoses - secondary ICD10: Alcoholic cirrhosis of liver	No
56	41200 value L541	7.02e-05	0.358 [0.181, 0.534]	-	BINARY	Operative procedures - main OPCS: Percutaneous transluminal angioplasty of iliac artery	No
57	41202 value C341	8.74e-05	0.246 [0.123, 0.368]	0.132 [-0.175, 0.431]	BINARY	Diagnoses - main ICD10: Malignant neoplasm of bronchus and lung, Upper lobe, bronchus or lung	No
58	1518	9.20e-05	0.032 [0.016, 0.048]	-0.000 [-0.015, 0.014]	ORDERED	Hot drink temperature	{Very hot … warm}
59	30070	9.44e-05	0.015 [0.008, 0.023]	-0.001 [-0.008, 0.005]	LINEAR	Red blood cell (erythrocyte) distribution width	-
60	41202 value I739	1.04e-04	0.255 [0.125, 0.383]	-0.034 [-0.360, 0.281]	BINARY	Diagnoses - main ICD10: Peripheral vascular disease, unspecified	No
61	41202 value C621	1.13e-04	1.711 [0.886, 2.652]	0.871 [0.100, 1.658]	BINARY	Diagnoses - main ICD10: Descended testis	No
62	41200 value L544	1.24e-04	0.443 [0.215, 0.669]	-	BINARY	Operative procedures - main OPCS: Percutaneous transluminal insertion of stent into iliac artery	No
63	41202 value C349	1.40e-04	0.208 [0.100, 0.314]	0.063 [-0.173, 0.294]	BINARY	Diagnoses - main ICD10: Malignant neoplasm of bronchus and lung,Bronchus or lung, unspecified	No

64	41204 value I972	1.46e-04	1.242 [0.613, 1.906]	-0.063 [-0.684, 0.516]	BINARY	Diagnoses - secondary ICD10: Postmastectomy lymphoedema syndrome	No
65	2897	1.53e-04	0.019 [0.009, 0.029]	*EVER ONLY PHENO*	LINEAR	Age stopped smoking	-
66	20004 value 1129	1.57e-04	0.167 [0.080, 0.253]	0.062 [-0.039, 0.162]	BINARY	Operation code: throat/larynx surgery (incl tracheostomy)	No
67	41204 value F220	1.59e-04	0.827 [0.398, 1.259]	-0.031 [-0.558, 0.466]	BINARY	Diagnoses - secondary ICD10: Delusional disorder	No
68	6157 value 2	1.65e-04	0.077 [0.037, 0.117]	*EVER ONLY PHENO*	BINARY	Why stopped smoking: Doctor's advice	No
69	41204 value I739	1.80e-04	0.150 [0.071, 0.228]	-0.014 [-0.179, 0.147]	BINARY	Diagnoses - secondary ICD10: Peripheral vascular disease, unspecified	No
