# Supplementary material for: MR-pheWAS with stratification and interaction: Searching for the causal effects of smoking heaviness identified an effect on facial aging
Source: PLoS Genet. 2019 Oct 31;15(10):e1008353. doi: 10.1371/journal.pgen.1008353 (PMC6822717; doi:10.1371/journal.pgen.1008353)
Supplement: S5 Table — Total number of tests in whole sample = 23009. Bonferroni threshold = 0.05/23009 = 2.17x10^-6^. False discovery rate threshold = 0.05x48/23009 = 1.04x10^-4^. (PDF) [file pgen.1008353.s006.pdf]

Rank	Field ID [4]	P value	Association among whole sample	Association among ever smokers [1]	Association among never smokers [1]	Regression Type	Field description	Reference, baseline or ordinal categories [3]
1	2887	7.25e-74	0.194 [0.173, 0.215]	0.194 [0.173, 0.215]	*EVER ONLY PHENO*	ORDERED-LOGISTIC	Number of cigarettes previously smoked daily	*{3 quantity bands}*
2	20162	3.59e-73	0.084 [0.075, 0.093]	0.084 [0.075, 0.093]	*EVER ONLY PHENO*	LINEAR	Pack years adult smoking as proportion of life span exposed to smoking	-
3	20161	3.59e-70	0.081 [0.072, 0.090]	0.081 [0.072, 0.090]	*EVER ONLY PHENO*	LINEAR	Pack years of smoking	-
4	3456	1.26e-27	0.206 [0.169, 0.244]	0.206 [0.169, 0.244]	*EVER ONLY PHENO*	ORDERED-LOGISTIC	Number of cigarettes currently smoked daily (current cigarette smokers)	*{3 quantity bands}*
5	2644	2.54e-26	-0.108 [-0.128, -0.088]	-0.105 [-0.147, -0.063]	-0.056 [-0.099, -0.012]	LOGISTIC-BINARY	"Light smokers at least 100 smokes in lifetime"	No
6	3476	1.40e-22	0.180 [0.144, 0.216]	0.180 [0.144, 0.216]	*EVER ONLY PHENO*	ORDERED-LOGISTIC	Difficulty not smoking for 1 day	{Very easy … Very difficult}
7	1797	2.09e-15	-0.059 [-0.073, -0.044]	-0.060 [-0.082, -0.037]	-0.059 [-0.078, -0.040]	LOGISTIC-BINARY	Father still alive	No
8	3063	3.28e-14	-0.015 [-0.019, -0.011]	-0.029 [-0.035, -0.023]	-0.006 [-0.011, -0.001]	LINEAR	Forced expiratory volume in 1-second (FEV1)	-
9	20150	4.28e-14	-0.016 [-0.021, -0.012]	-0.034 [-0.040, -0.027]	-0.004 [-0.009, 0.002]	LINEAR	"Forced expiratory volume in 1-second (FEV1) Best measure"	-
10	3466	4.03e-13	-0.131 [-0.167, -0.096]	-0.131 [-0.167, -0.096]	*EVER ONLY PHENO*	ORDERED-LOGISTIC	Time from waking to first cigarette	*{Less than 5 minutes … Longer than 2 hours}*
11	20107 value 3	4.67e-13	0.069 [0.050, 0.088]	0.064 [0.036, 0.091]	0.075 [0.050, 0.100]	LOGISTIC-BINARY	Illnesses of father: Lung cancer	No
12	20107 value 6	7.52e-13	0.063 [0.045, 0.080]	0.071 [0.047, 0.096]	0.057 [0.033, 0.080]	LOGISTIC-BINARY	Illnesses of father: Chronic bronchitis/emphysema	No
13	20154	1.20e-11	-0.031 [-0.040, -0.022]	-0.067 [-0.083, -0.050]	-0.004 [-0.015, 0.007]	LINEAR	Forced expiratory volume in 1-second (FEV1) predicted percentage	-
14	1807	2.25e-11	-0.020 [-0.026, -0.014]	-0.020 [-0.029, -0.012]	-0.020 [-0.028, -0.013]	LINEAR	Father's age at death	-
15	41204 value J449	9.17e-11	0.130 [0.091, 0.169]	0.172 [0.129, 0.216]	-0.023 [-0.129, 0.080]	LOGISTIC-BINARY	Diagnoses - secondary ICD10: Chronic obstructive pulmonary disease, unspecified	No
16	20110 value 3	1.82e-10	0.087 [0.060, 0.113]	0.082 [0.043, 0.122]	0.090 [0.054, 0.126]	LOGISTIC-BINARY	Illnesses of mother: Lung cancer	No
17	41204 value J439	1.84e-10	0.264 [0.182, 0.345]	0.342 [0.256, 0.427]	-0.350 [-0.635, -0.077]	LOGISTIC-BINARY	Diagnoses - secondary ICD10: Emphysema, unspecified	No
18	20110 value 6	3.98e-10	0.072 [0.049, 0.094]	0.050 [0.017, 0.083]	0.090 [0.059, 0.121]	LOGISTIC-BINARY	Illnesses of mother: Chronic bronchitis/emphysema	No
19	20111 value 6	2.51e-09	0.106 [0.071, 0.141]	0.131 [0.084, 0.178]	0.081 [0.029, 0.133]	LOGISTIC-BINARY	Illnesses of siblings: Chronic bronchitis/emphysema	No
20	3062	7.68e-09	-0.011 [-0.015, -0.007]	-0.017 [-0.023, -0.011]	-0.008 [-0.013, -0.003]	LINEAR	Forced vital capacity (FVC)	-
21	20151	1.36e-08	-0.012 [-0.016, -0.008]	-0.020 [-0.026, -0.014]	-0.006 [-0.012, -0.001]	LINEAR	Forced vital capacity (FVC) Best measure	-
22	6149 value 6	8.41e-08	0.039 [0.025, 0.053]	0.060 [0.041, 0.079]	0.020 [-0.002, 0.042]	LOGISTIC-BINARY	Mouth/teeth dental problems: Dentures	No
23	2926	9.52e-08	0.054 [0.034, 0.074]	0.054 [0.034, 0.074]	*EVER ONLY PHENO*	ORDERED-LOGISTIC	Number of unsuccessful stop-smoking attempts	*{3 quantity bands}*
24	22507	3.65e-07	0.043 [0.026, 0.059]	0.042 [0.025, 0.059]	0.009 [-0.071, 0.089]	LINEAR	Age of stopping smoking	-
25	6157 value 1	8.09e-07	0.080 [0.048, 0.112]	0.079 [0.048, 0.111]	*EVER ONLY PHENO*	LOGISTIC-BINARY	Why stopped smoking: Illness or ill health	No
26	1757	1.53e-06	0.030 [0.018, 0.042]	0.060 [0.042, 0.078]	0.004 [-0.012, 0.021]	ORDERED-LOGISTIC	Facial ageing	*{Younger than you are … Older than you are}*
Bonferroni Threshold								
28	30050	2.67e-06	0.012 [0.007, 0.017]	0.014 [0.006, 0.022]	0.012 [0.005, 0.019]	LINEAR	Mean corpuscular haemoglobin	-
27	1835	2.69e-06	-0.030 [-0.042, -0.017]	-0.014 [-0.033, 0.005]	-0.042 [-0.058, -0.025]	LOGISTIC-BINARY	Mother still alive	No
29	20004 value 1491	2.93e-06	0.104 [0.060, 0.148]	0.120 [0.053, 0.187]	0.090 [0.032, 0.148]	LOGISTIC-BINARY	Operation code: squint correction	No
30	22508	8.34e-06	0.164 [0.092, 0.236]	0.165 [0.093, 0.237]	*EVER ONLY PHENO*	LINEAR	Amount of tobacco currently smoked	-
31	41202 value C621	1.20e-05	1.259 [0.706, 1.841]	1.711 [0.886, 2.652]	0.871 [0.100, 1.658]	LOGISTIC-BINARY	Diagnoses - main ICD10: Descended testis	No
32	30040	1.21e-05	0.011 [0.006, 0.017]	0.017 [0.009, 0.024]	0.008 [0.001, 0.015]	LINEAR	Mean corpuscular volume	-
33	2907	1.84e-05	-0.046 [-0.067, -0.025]	-0.046 [-0.067, -0.025]	*EVER ONLY PHENO*	LOGISTIC-BINARY	Ever stopped smoking for 6+ months	No

34	41202 value J441	2.45e-05	0.223 [0.119, 0.326]	0.260 [0.152, 0.368]	-0.052 [-0.471, 0.348]	LOGISTIC-BINARY	Diagnoses - main ICD10: Chronic obstructive pulmonary disease with acute exacerbation, unspecified	No
35	20160	2.98e-05	-0.022 [-0.033, -0.012]	-	-0.019 [-0.035, -0.004]	LOGISTIC-BINARY	Ever smoked	No
36	25062	3.09e-05	0.063 [0.033, 0.092]	0.081 [0.033, 0.128]	0.050 [0.012, 0.087]	LINEAR	Mean FA in corticospinal tract on FA skeleton (right)	-
37	40001 value C349	3.20e-05	0.195 [0.103, 0.286]	0.225 [0.126, 0.323]	0.059 [-0.212, 0.323]	LOGISTIC-BINARY	Underlying (primary) cause of death: ICD10: Malignant neoplasm of bronchus and lung, unspecified	No
38	25356	3.99e-05	0.062 [0.032, 0.091]	0.089 [0.041, 0.136]	0.044 [0.007, 0.082]	LINEAR	Mean ICVF in superior cerebellar peduncle on FA skeleton (right)	-
39	41204 value N130	4.41e-05	1.220 [0.645, 1.823]	1.291 [0.441, 2.210]	1.177 [0.395, 2.008]	LOGISTIC-BINARY	Diagnoses - secondary ICD10: Hydronephrosis with ureteropelvic junction obstruction	No
40	1359	4.57e-05	-0.020 [-0.030, -0.010]	-0.030 [-0.044, -0.015]	-0.012 [-0.025, 0.001]	ORDERED-LOGISTIC	Poultry intake	{Never ... Once or more daily}
41	41200 value L541	4.88e-05	0.359 [0.185, 0.531]	0.358 [0.181, 0.534]	-	LOGISTIC-BINARY	Operative procedures - main OPCS: Percutaneous transluminal angioplasty of iliac artery	No
42	5096	7.54e-05	-0.022 [-0.033, -0.011]	-0.021 [-0.037, -0.004]	-0.022 [-0.037, -0.008]	LINEAR	3mm weak meridian (left)	-
43	6152 value 6	8.50e-05	0.080 [0.040, 0.120]	0.117 [0.069, 0.164]	0.018 [-0.059, 0.094]	LOGISTIC-BINARY	Blood clot, DVT, bronchitis, emphysema, asthma, rhinitis, eczema, allergy diagnosed by doctor: Emphysema/chronic bronchitis	
44	41210 value Z863	8.51e-05	0.273 [0.136, 0.409]	0.288 [0.080, 0.492]	0.258 [0.074, 0.440]	LOGISTIC-BINARY	Operative procedures - secondary OPCS: Tarsometatarsal joint	No
45	41204 value Z720	9.03e-05	0.061 [0.031, 0.092]	0.074 [0.042, 0.106]	0.020 [-0.140, 0.176]	LOGISTIC-BINARY	Diagnoses - secondary ICD10: tobacco use	No
46	5098	9.79e-05	-0.023 [-0.035, -0.012]	-0.018 [-0.036, -0.001]	-0.027 [-0.042, -0.011]	LINEAR	6mm weak meridian (right)	-
47	20004 value 1104	9.94e-05	0.358 [0.177, 0.537]	0.508 [0.300, 0.713]	-0.089 [-0.484, 0.286]	LOGISTIC-BINARY	Operation code: aortic aneurysm/repair or stent	No
48	25261	1.00e-04	-0.113 [-0.170, -0.056]	-0.164 [-0.256, -0.073]	-0.079 [-0.151, -0.006]	ORDERED-LOGISTIC	Mean L2 in superior cerebellar peduncle on FA skeleton (left)	-
