# Supplementary material for: MR-pheWAS with stratification and interaction: Searching for the causal effects of smoking heaviness identified an effect on facial aging
Source: PLoS Genet. 2019 Oct 31;15(10):e1008353. doi: 10.1371/journal.pgen.1008353 (PMC6822717; doi:10.1371/journal.pgen.1008353)
Supplement: S6 Table — Step 1: Rank results by association strength among whole sample, identifies 48 results (S4 Table), of which 36 have an interaction P value for use in step 2. Step 2: Rank results by strength of interaction of ever versus never smokers. Bonferroni threshold = 0.05/36 = 1.39x10^-3^. False discovery rate threshold = 0.05x9/36 = 1.25x10^-2^. 1 Linear: associations are the SD difference in inverse rank normal transformed outcome for each additional smoking-increasing allele of rs16969968. Ordered: Associations are the log odds of a higher outcome group for each additional smoking-increasing allele of rs16969968. Binary: Associations are the log odds of comparison versus baseline outcome group for each additional smoking-increasing allele of rs16969968. 2 Strength of interaction between ever versus never smokers using meta regression (metan command in Stata). 3 Information on categories for multinomial, ordinal and binary regression results: reference category for multinomial regression results, baseline category for binary logistic regression results, and category ordering for ordered logistic regression results. For example, {Younger than you are … Older than you are}” for field 1757 means that there are categories ranging from “Younger than you are” to “Older than you are” where the former is coded with the smallest value, and the latter with the largest value. 4 In addition to the field ID, this column also contains the reference value for multinomial regression results, and the field value for which a binary variable was generated for categorical (multiple) fields. (PDF) [file pgen.1008353.s007.pdf]

Rank	Field ID [4]	Interaction P value [2]	Association among ever smokers [1]	Association among never smokers [1]	Regression Type	Field description	Reference, baseline or ordinal categories [3]
1	20150	7.61e-12	-0.034 [-0.040, -0.027]	-0.004 [-0.009, 0.002]	LINEAR	Forced expiratory volume in 1-second (FEV1), Best measure	-
2	20154	4.49e-10	-0.067 [-0.083, -0.050]	-0.004 [-0.015, 0.007]	LINEAR	Forced expiratory volume in 1-second (FEV1), predicted percentage	-
3	3063	1.06e-08	-0.029 [-0.035, -0.023]	-0.006 [-0.011, -0.001]	LINEAR	Forced expiratory volume in 1-second (FEV1)	-
4	41204 value J439	3.54e-06	0.342 [0.256, 0.427]	-0.350 [-0.635, -0.077]	BINARY	Diagnoses - secondary ICD10: Emphysema, unspecified	No
5	1757	7.71e-06	0.060 [0.042,0.078]	0.004 [-0.012, 0.021]	ORDERED	Facial ageing	*{Younger than you are … Older than you are}*
6	41204 value J449	6.76e-04	0.172 [0.129, 0.216]	-0.023 [-0.129, -0.080]	BINARY	Diagnoses - secondary ICD10: Chronic obstructive pulmonary disease, unspecified	No
Bonferroni threshold							
7	20151	1.23e-03	-0.020 [-0.026, -0.014]	-0.006 [-0.012, -0.001]	LINEAR	Forced vital capacity (FVC), Best measure	-
8	6149 value 6	6.71e-03	0.060 [0.041, 0.079]	0.020 [-0.002, 0.042]	BINARY	Mouth/teeth dental problems: Dentures	No
9	20004 value 1104	7.41e-03	0.508 [0.300, 0.713]	-0.089 [-0.484, 0.286]	BINARY	Operation code: aortic aneurysm/repair or stent	No
