# Supplementary figures and images for: MR-pheWAS with stratification and interaction: Searching for the causal effects of smoking heaviness identified an effect on facial aging
Source: PLoS Genet. 2019 Oct 31;15(10):e1008353. doi: 10.1371/journal.pgen.1008353 (PMC6822717; doi:10.1371/journal.pgen.1008353)

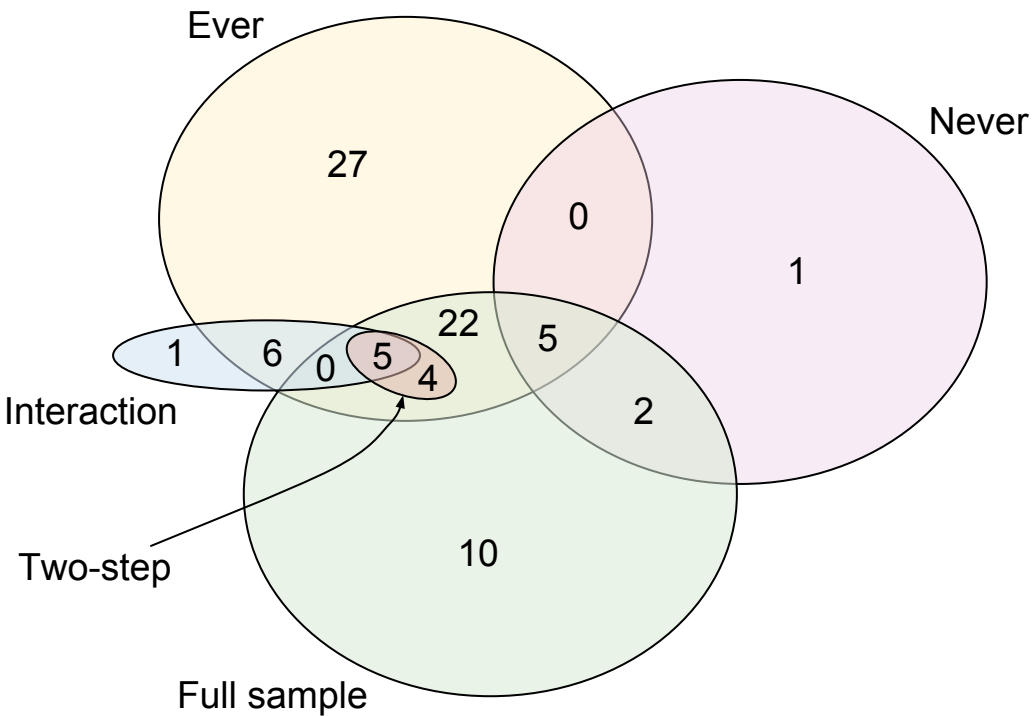

Supplement: S1 Fig — (PDF) [file pgen.1008353.s008.pdf]

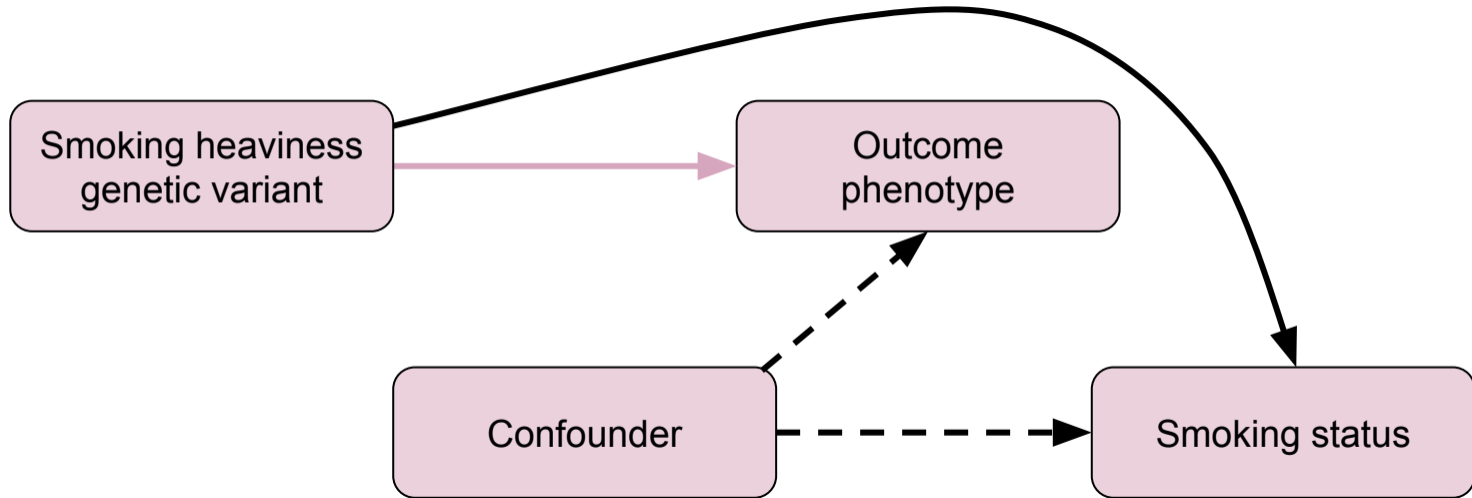

Supplement: S2 Fig — In our UK Biobank sample the smoking heaviness genetic variant is estimated to affect both smoking status and the identified outcome phenotypes. If a confounder exists that affects both smoking status and a given outcome then smoking status is a collider and stratifying on smoking status may cause collider bias–bias in the estimated effect of the genetic variant on the outcome phenotype. An exception to this is when the genetic variant for smoking heaviness does not affect the outcome, and they act additively on smoking status (i.e. there is no additive interaction on the log-probability scale). In this case, collider bias would not occur. (PDF) [file pgen.1008353.s009.pdf]

## Ever smokers

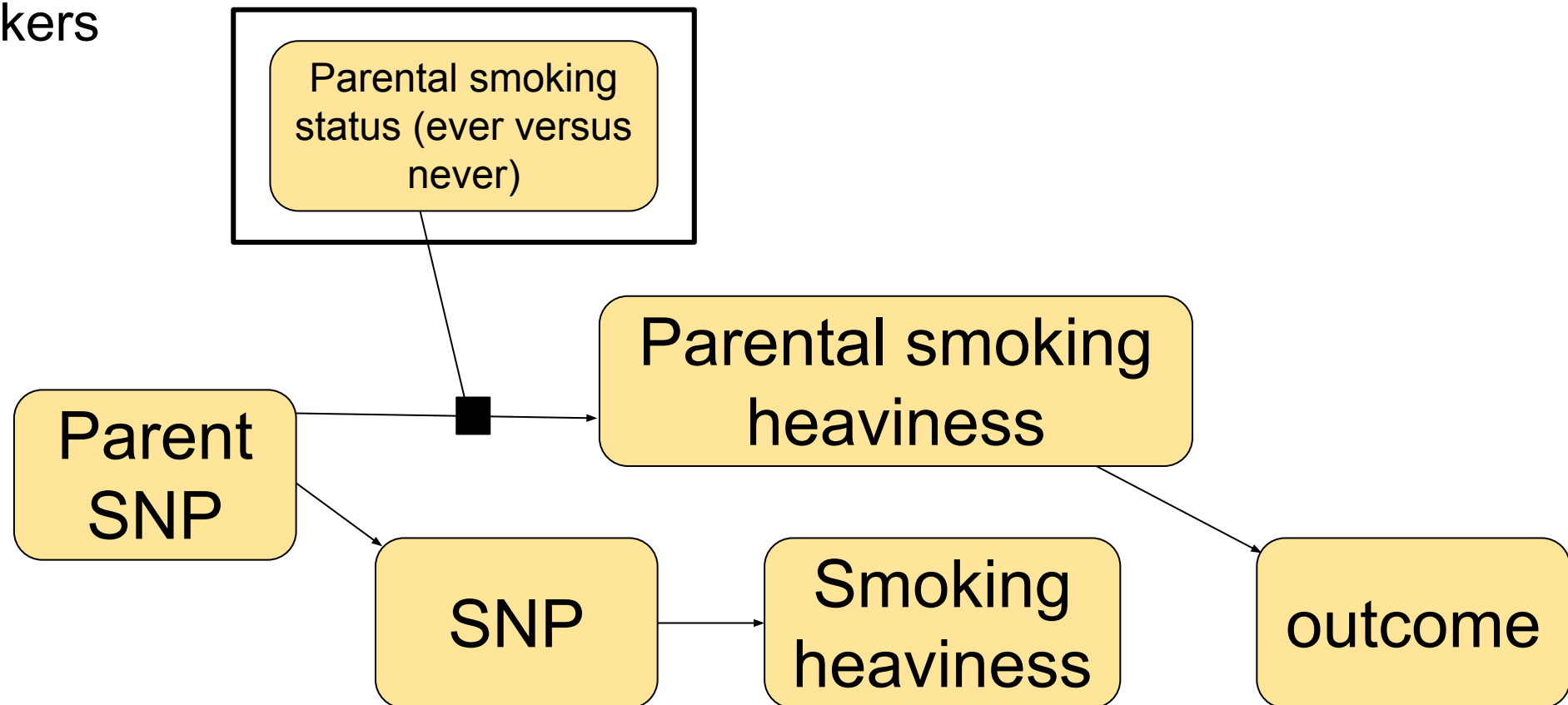

## Never smokers

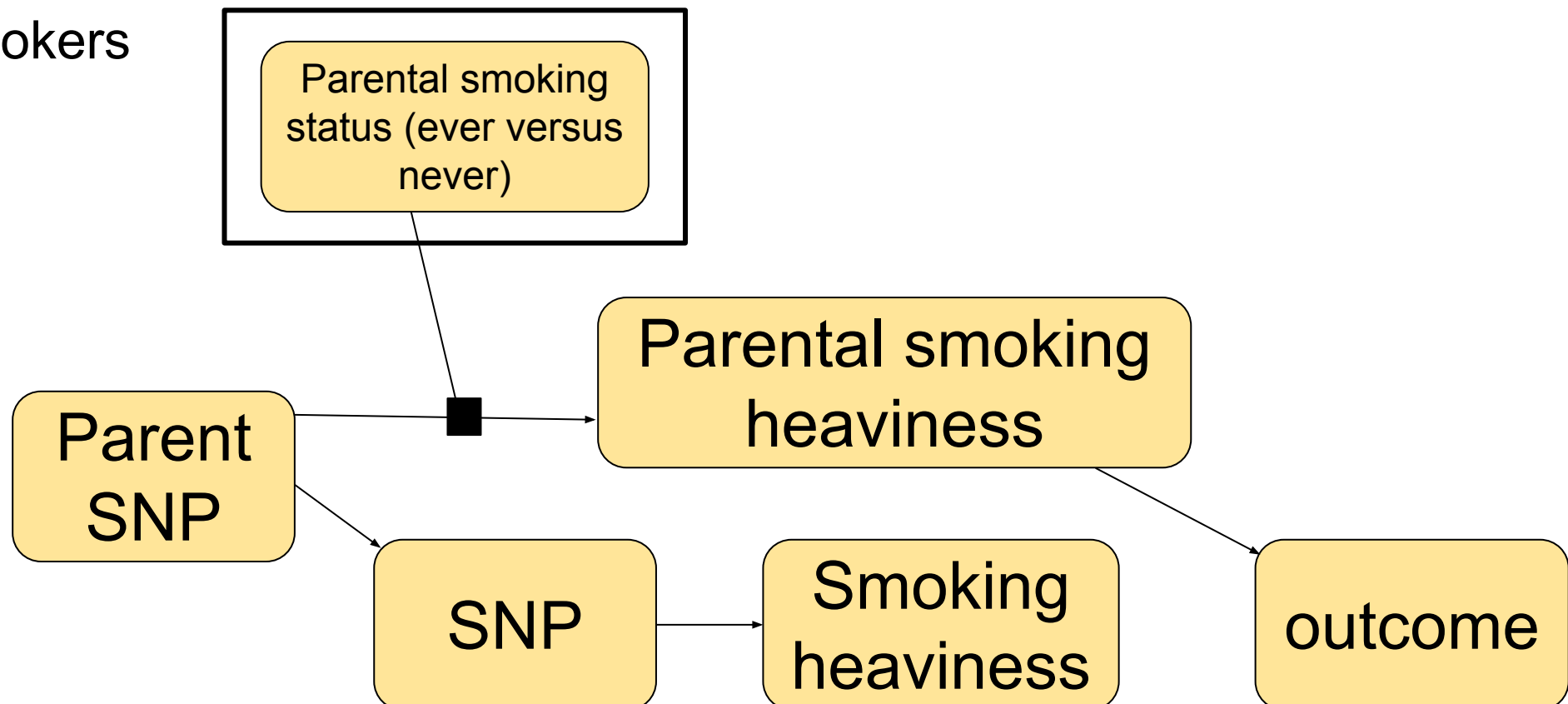

Supplement: S7 Fig — In this figure we show two directed acyclic graphs (DAGs), after stratification by ever versus never smokers (of the UK Biobank participants). We assume an effect of parental smoking heaviness on the UK Biobank participants outcome. This alternative pathway between the UK Biobank participant's SNP and the outcome (via parental SNP and parental smoking heaviness) is the same in UK Biobank ever and never smokers. It is not horizontal pleiotropy because there is no causal effect of the SNP (or more precisely the genetic variation for which the SNP is tagging) on the outcome–it is the parental genetic variation that affects the outcome. Note also that this assumes participant smoking status is independent of parental smoking status, otherwise the extent to which this alternative pathway manifests would vary in UK Biobank participant ever versus never smokers. (PDF) [file pgen.1008353.s014.pdf]
