# Supplementary material for: MR-pheWAS with stratification and interaction: Searching for the causal effects of smoking heaviness identified an effect on facial aging
Source: PLoS Genet. 2019 Oct 31;15(10):e1008353. doi: 10.1371/journal.pgen.1008353 (PMC6822717; doi:10.1371/journal.pgen.1008353)

a) $OR_{conf,si}$ = 10, positive effect of confounder on outcome

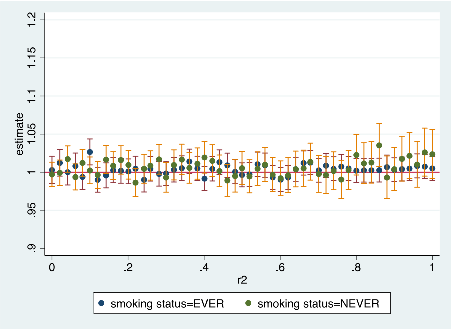

b) $OR_{conf,si}$ = 20, positive effect of confounder on outcome

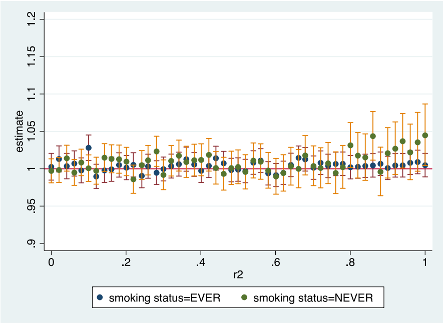

c) $OR_{conf,si}$ = 50, positive effect of confounder on outcome

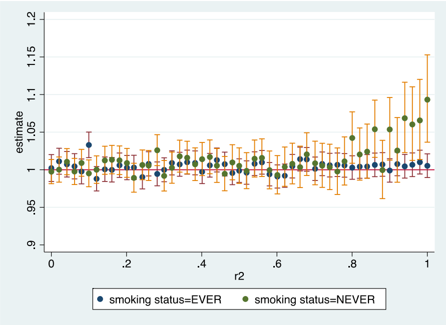

d) $OR_{conf,si}$ = 100, positive effect of confounder on outcome

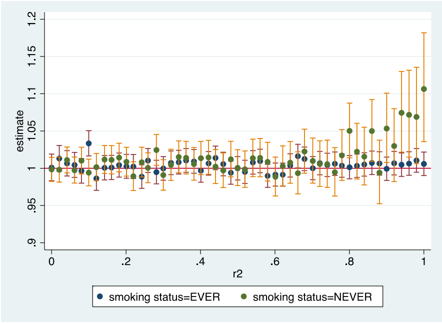

e) $OR_{conf,si}$ = 10, negative effect of confounder on outcome

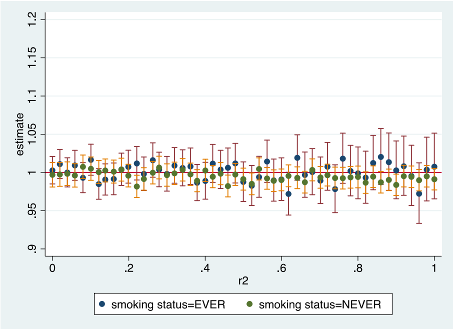

f) $OR_{conf,si}$ = 20, negative effect of confounder on outcome

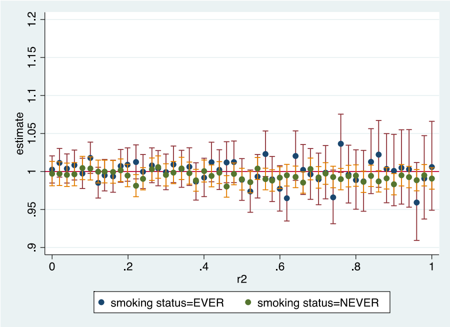

g) $OR_{conf,si}$ = 50, negative effect of confounder on outcome

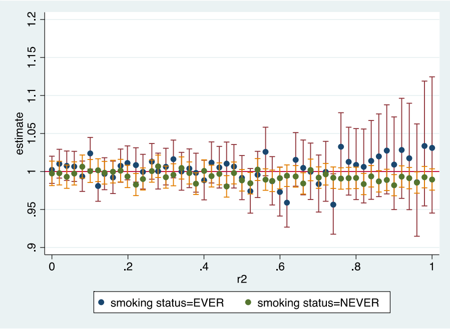

h) $OR_{conf,si}$ = 100, negative effect of confounder on outcome

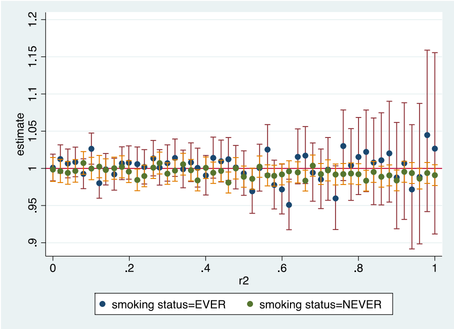

Supplement: S3 Fig — ORconf,si is the odds ratio of the confounder on smoking status, i.e. the change of odds of being an ever versus never smoker for a 1 standard deviation increase in confounder. r2 is proportion of the variance of the continuous facial aging phenotype (underlying the categorical facial aging outcome) that is explained by the confounder. a-d: positive effect of confounder on outcome, with OR of the confounder on smoking status of 10 (a), 20 (b), 50 (c) and 100 (d). e-h: negative effect of confounder on outcome, with OR of the confounder on smoking status of 10 (e), 20 (f), 50 (g) and 100 (h). (PDF) [file pgen.1008353.s010.pdf]
