# Supplementary material for: MR-pheWAS with stratification and interaction: Searching for the causal effects of smoking heaviness identified an effect on facial aging
Source: PLoS Genet. 2019 Oct 31;15(10):e1008353. doi: 10.1371/journal.pgen.1008353 (PMC6822717; doi:10.1371/journal.pgen.1008353)

a) $OR_{conf,si} = 10$, positive effect of confounder on outcome

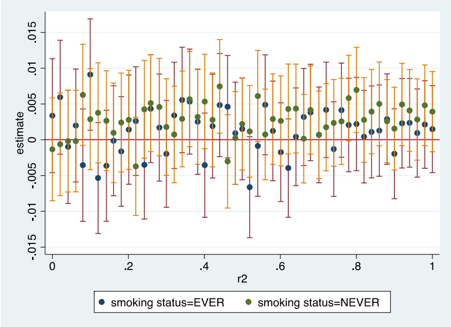

b) $OR_{conf,si} = 20$, positive effect of confounder on outcome

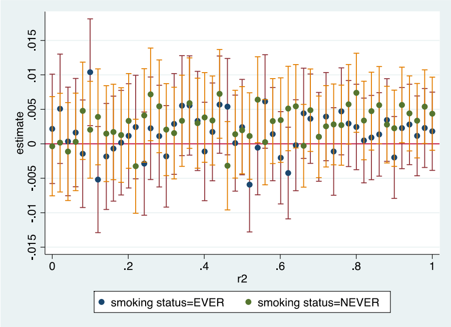

c) $OR_{conf,si} = 50$, positive effect of confounder on outcome

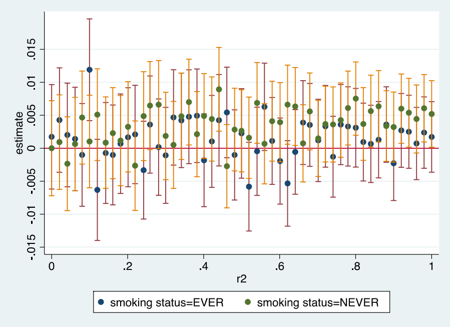

d) $OR_{conf,si} = 100$, positive effect of confounder on outcome

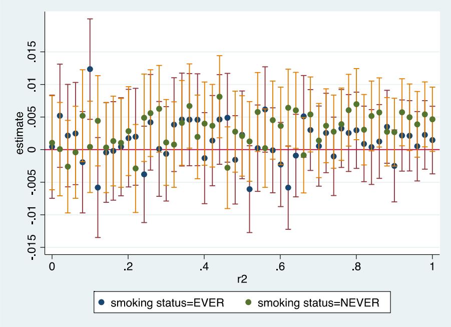

e) $OR_{conf,si} = 10$, negative effect of confounder on outcome

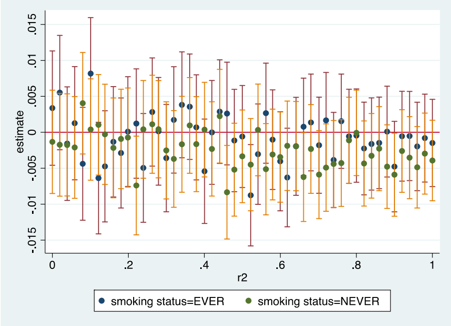

f) $OR_{conf,si} = 20$, negative effect of confounder on outcome

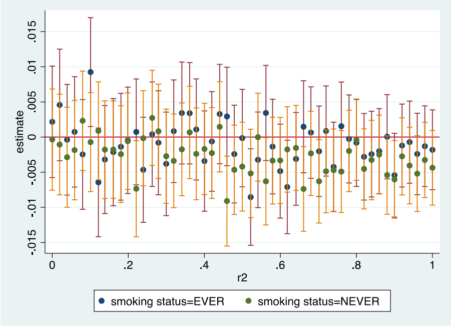

g) $OR_{conf,si} = 50$, negative effect of confounder on outcome

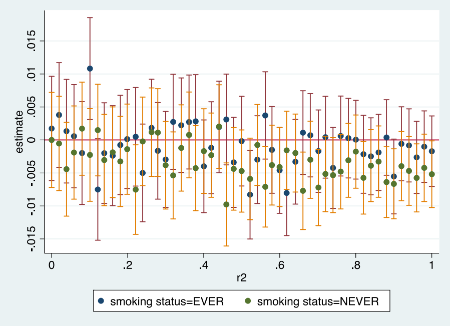

h) $OR_{conf,si} = 100$, negative effect of confounder on outcome

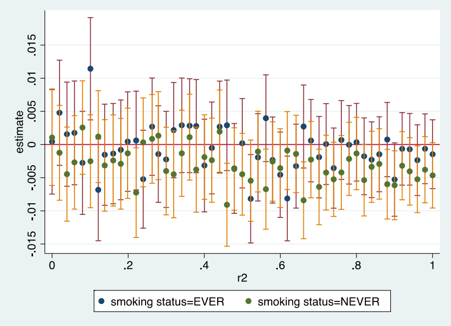

Supplement: S4 Fig — ORconf,si is the odds ratio of the confounder on smoking status, i.e. that change of odds of being an ever versus never smoker for a 1 standard deviation increase in confounder. r2 is proportion of the variance of the continuous phenotype that is explained by the confounder. a-d: positive effect of confounder on outcome, with OR of the confounder on smoking status of 10 (a), 20 (b), 50 (c) and 100 (d). e-h: negative effect of confounder on outcome, with OR of the confounder on smoking status of 10 (e), 20 (f), 50 (g) and 100 (h). (PDF) [file pgen.1008353.s011.pdf]
