# Supplementary material for: MR-pheWAS with stratification and interaction: Searching for the causal effects of smoking heaviness identified an effect on facial aging
Source: PLoS Genet. 2019 Oct 31;15(10):e1008353. doi: 10.1371/journal.pgen.1008353 (PMC6822717; doi:10.1371/journal.pgen.1008353)

a) Categorical facial aging outcome, positive effect of confounder on outcome

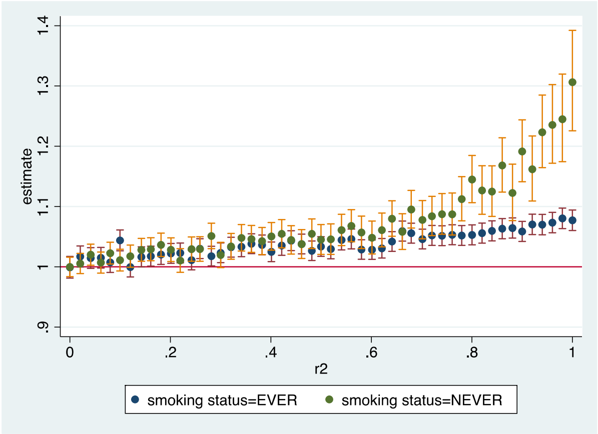

b) Continuous outcome, positive effect of confounder on outcome

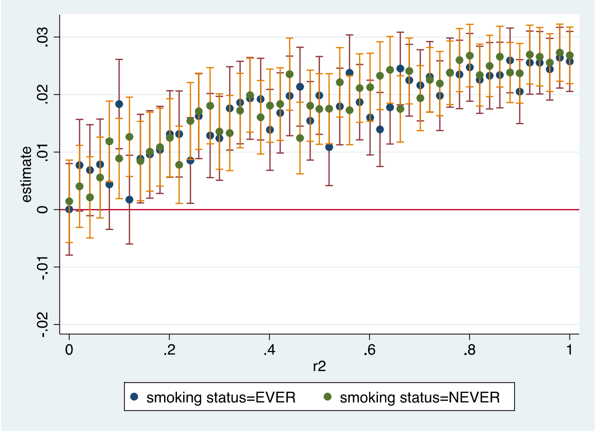

c) Categorical facial aging outcome, negative effect of confounder on outcome

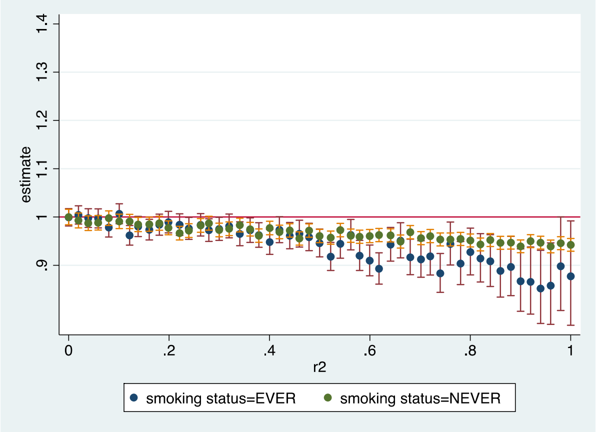

d) Continuous outcome, negative effect of confounder on outcome

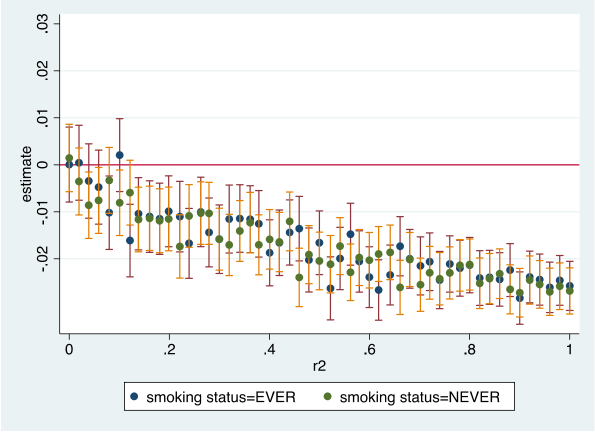

Supplement: S5 Fig — These simulations assume a large (and hence unlikely) effect of the confounder on smoking status (odds ratio = 100; i.e. we are assuming a 1 standard deviation change of the confounder causes a 100 higher odds of being an ever smoker). Furthermore, we assume a 1 dosage increase in SNP causes a 0.8 lower odds of being an ever smoker (i.e. we have made this more extreme than we see in UK Biobank for illustration purposes). (a) Categorical facial aging outcome, positive effect of confounder on outcome. (b) Continuous outcome, positive effect of confounder on outcome. (c) Categorical facial aging outcome, negative effect of confounder on outcome. (d) Continuous outcome, negative effect of confounder on outcome. In plots (a) and (c) r2 is proportion of the variance of the continuous facial aging phenotype (underlying the categorical facial aging outcome) that is explained by the confounder. In plots (b) and (d) r2 is proportion of the variance of the continuous phenotype that is explained by the confounder. (PDF) [file pgen.1008353.s012.pdf]
