# Supplementary material for: MR-pheWAS with stratification and interaction: Searching for the causal effects of smoking heaviness identified an effect on facial aging
Source: PLoS Genet. 2019 Oct 31;15(10):e1008353. doi: 10.1371/journal.pgen.1008353 (PMC6822717; doi:10.1371/journal.pgen.1008353)
Supplement: S6 Fig — Solid black boxes indicate an interaction. Box around smoking status variable indicates that this variable is conditioned upon (i.e. we stratify on ever versus never smokers). Please see code in the project’s GitHub repository [http://github.com/MRCIEU/PHESANT-MR-pheWAS-smoking/] for simulations of these scenarios. a) Our hypothesised pathway, where the genetic variant affects the outcome via smoking heaviness. b) Independent effects of the SNP and smoking status on the outcome are not problematic–the effect estimates in smoking status strata (ever versus never) are still consistent. c) If there is only an effect on the outcome via smoking status, rather than smoking heaviness, then within smoking status strata there is no association between the SNP and the outcome. d) An interaction of smoking status along the pathway between the SNP and outcome would also give an interaction of the effects of the SNP across smoking status strata. (PDF) [file pgen.1008353.s013.pdf]

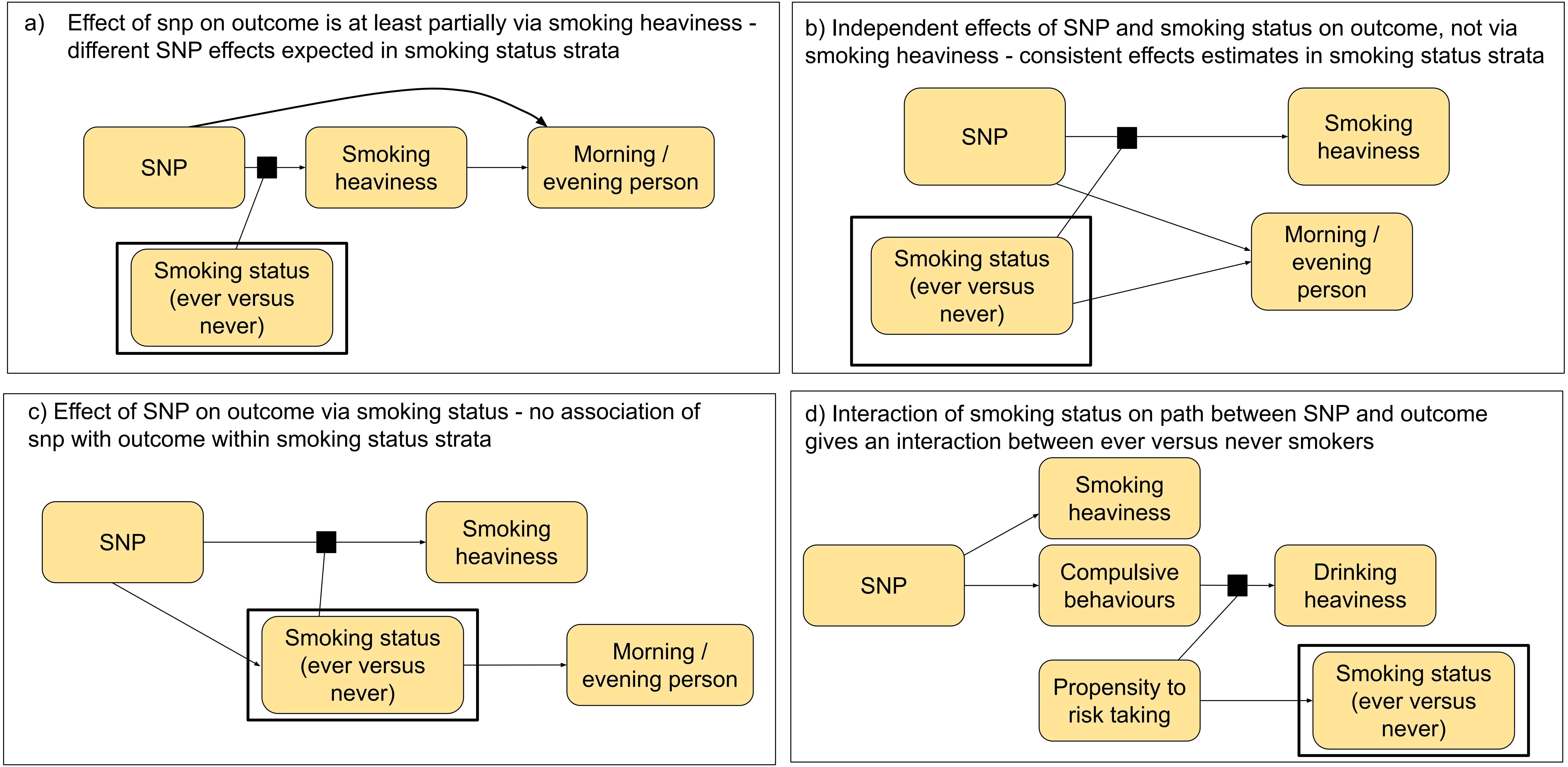

a) Effect of snp on outcome is at least partially via smoking heaviness - different SNP effects expected in smoking status strata
SNP
Smoking heaviness
Morning / evening person
Smoking status (ever versus never)
b) Independent effects of SNP and smoking status on outcome, not via smoking heaviness - consistent effects estimates in smoking status strata
SNP
Smoking heaviness
Morning / evening person
Smoking status (ever versus never)
c) Effect of SNP on outcome via smoking status - no association of snp with outcome within smoking status strata
SNP
Smoking heaviness
Smoking status (ever versus never)
Morning / evening person
d) Interaction of smoking status on path between SNP and outcome gives an interaction between ever versus never smokers
Smoking heaviness
SNP
Compulsive behaviours
Drinking heaviness
Propensity to risk taking
Smoking status (ever versus never)
